# Galanin Neurons Unite Sleep Homeostasis and α2-Adrenergic Sedation

**DOI:** 10.1016/j.cub.2019.07.087

**Published:** 2019-10-07

**Authors:** Ying Ma, Giulia Miracca, Xiao Yu, Edward C. Harding, Andawei Miao, Raquel Yustos, Alexei L. Vyssotski, Nicholas P. Franks, William Wisden

**Affiliations:** 1 Department of Life Sciences, Imperial College, London SW7 2AZ, UK; 2 UK Dementia Research Institute, Imperial College, London SW7 2AZ, UK; 3 Institute of Neuroinformatics, University of Zurich and ETH Zurich, Zurich 8057, Switzerland

**Keywords:** sleep homeostasis, dexmedetomidine, galanin, body temperature, preoptic hypothalamus, sedation, NREM, torpor

## Abstract

Our urge to sleep increases with time spent awake, until sleep becomes inescapable. The sleep following sleep deprivation is longer and deeper, with an increased power of delta (0.5**–**4 Hz) oscillations, a phenomenon termed sleep homeostasis [1, 2, 3, 4]. Although widely expressed genes regulate sleep homeostasis [1, 4, 5, 6, 7, 8, 9, 10] and the process is tracked by somnogens and phosphorylation [1, 3, 7, 11, 12, 13, 14], at the circuit level sleep homeostasis has remained mysterious. Previously, we found that sedation induced with α2-adrenergic agonists (e.g., dexmedetomidine) and sleep homeostasis both depend on the preoptic (PO) hypothalamus [15, 16]. Dexmedetomidine, increasingly used for long-term sedation in intensive care units [17], induces a non-rapid-eye-movement (NREM)-like sleep but with undesirable hypothermia [18, 19]. Within the PO, various neuronal subtypes (e.g., GABA/galanin and glutamate/NOS1) induce NREM sleep [20, 21, 22] and concomitant body cooling [21, 22]. This could be because NREM sleep’s restorative effects depend on lower body temperature [23, 24]. Here, we show that mice with lesioned PO galanin neurons have reduced sleep homeostasis: in the recovery sleep following sleep deprivation there is a diminished increase in delta power, and the mice catch up little on lost sleep. Furthermore, dexmedetomidine cannot induce high-power delta oscillations or sustained hypothermia. Some hours after dexmedetomidine administration to wild-type mice there is a rebound in delta power when they enter normal NREM sleep, reminiscent of emergence from torpor. This delta rebound is reduced in mice lacking PO galanin neurons. Thus, sleep homeostasis and dexmedetomidine-induced sedation require PO galanin neurons and likely share common mechanisms.

## Highlights

• This is the first identification of a cell type underlying sleep homeostasis
• Preoptic galanin neurons are essential for sleep homeostasis
• Galanin neurons mediate the sedative and hypothermic actions of dexmedetomidine
• Dexmedetomidine causes an EEG delta power rebound dependent on galanin neurons

## Graphical Abstract

**Figure:**
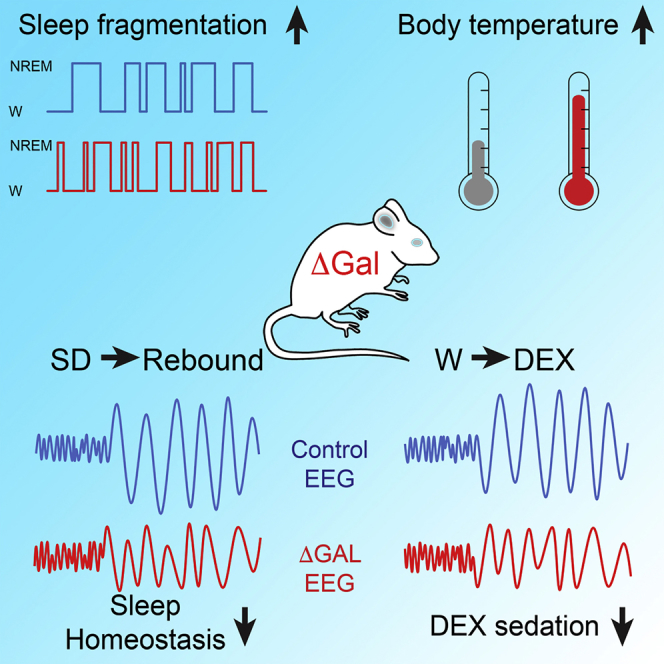

## Teaser

Sleep deprivation increases the urge to sleep. The subsequent sleep rebound is termed sleep homeostasis. Ma et al. find that sleep homeostasis, and its concomitant body cooling, is controlled by preoptic galanin neurons. These same neurons are also needed for α2-adrenergic drugs, such as dexmedetomidine, to induce sedation and cooling.

## Results and Discussion

### Ablating Galanin Neurons in the PO Hypothalamus

Ablating preoptic (PO) subnuclei produces insomnia and higher-amplitude diurnal oscillations in body temperature [25]. To selectively ablate PO^Gal^ neurons, we bilaterally injected an AAV mixture expressing Cre-activatable caspase-3 (AAV-*FLEX-CASP3*) and GFP (AAV*-FLEX-GFP*) transgenes into the lateral preoptic (LPO) area of *Gal-Cre* mice, generating *LPO-*Δ*Gal* mice (Figures S1A–S1D). (Note: in the *Gal-Cre* mouse line [26], 95% of PO galanin-expressing neurons co-express Cre, and 95% of PO Cre-expressing cells co-express galanin [27].) As controls, *Gal-Cre* mice were injected only with AAV*-FLEX-GFP* to generate *LPO-Gal-GFP* mice (Figure S1A). In the *LPO*-Δ*Gal* mouse group, immunohistochemistry with GFP antibodies showed that, after 5 weeks, the AAV*-FLEX-CASP3* injections eliminated ∼98% of LPO^Gal^ cells, as compared with *LPO-Gal-GFP* littermate controls (Figures S1B–S1D). Numbers of parvalbumin-expressing cells (a neuronal population not expressing galanin [28]) in LPO were unaffected by caspase deletion of galanin neurons (Figure S1E), implying that the caspase selectively killed galanin neurons.

### PO Galanin Neurons Are Needed for Consolidated NREM Sleep

To induce non-rapid-eye-movement (NREM) sleep, PO GABAergic neurons are believed to inhibit wake-promoting histamine neurons in the posterior hypothalamus [20, 29, 30] and express galanin [31]; indeed, galanin reduces the firing rate of histamine neurons [32]. There are, however, conflicting data on the consequences of activating PO galanin neurons. Optogenetic stimulation of PO galanin neuron soma at 10 Hz produced wakefulness [30]; however, lower stimulation frequencies (0.5–4 Hz) induced NREM sleep [21]. Chemogenetic activation of LPO galanin neurons also induced NREM sleep [21]—the authors of [21] found opto-stimulation frequencies above 8 Hz induced conduction block and inhibited PO galanin neurons, producing wake [21]. On the other hand, given that galanin neuronal subtypes exist [28], some could produce sleep, others wake.

**Figure 1:**
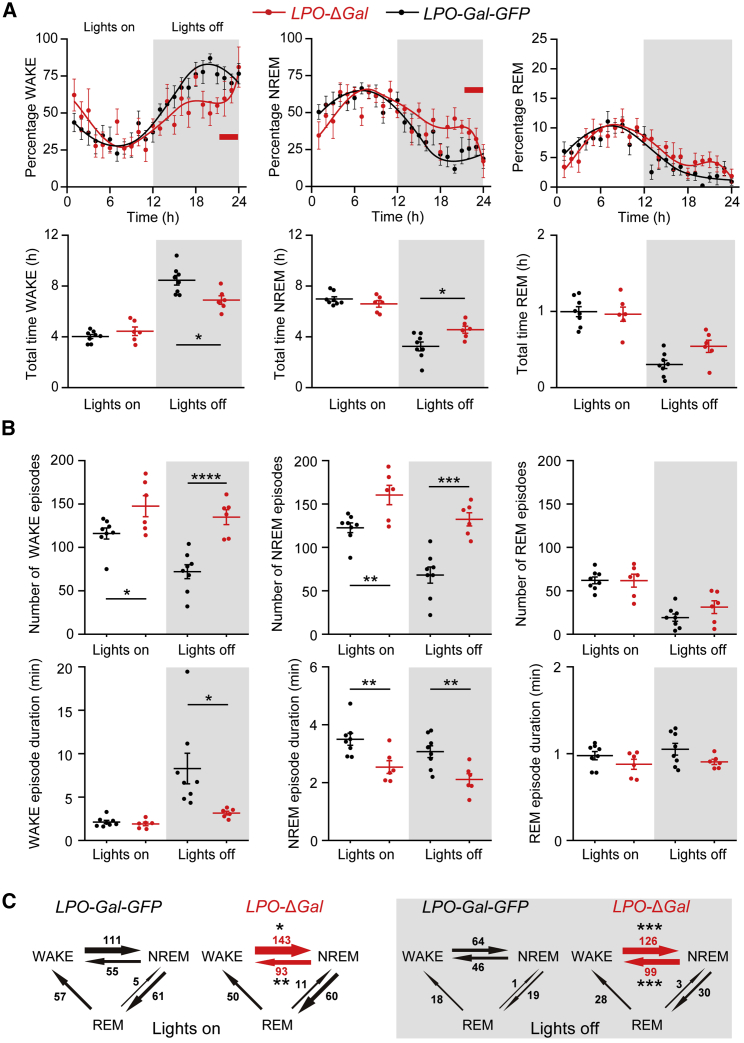
Ablation of Galanin Neurons in the LPO Caused Profound Fragmentation in Sleep Architecture (A) Ablation of galanin neurons (see Figure S1) caused a modest reduction in total WAKE time (^∗^p < 0.05, unpaired two-tailed t test) and an increase in total NREM time (^∗^p < 0.05, unpaired two-tailed t test) during “lights off,” but no change during “lights on.” The amount of REM sleep was unaffected. (B) Sleep architecture was highly fragmented by galanin neuron ablation. The number of WAKE and NREM episodes increased markedly, whereas their durations were shortened. These effects were most marked during lights off. The numbers of REM episodes and their durations were not affected. Example EEG and electromyogram (EMG) spectra are shown in Figure S2. (C) The number of WAKE-to-NREM and NREM-to-WAKE transitions was significantly altered, but transitions between other vigilance states did not change (*LPO-Gal-GFP*, n = 8; *LPO-*Δ*Gal*, n = 6). ^∗^p < 0.05, ^∗∗^p < 0.01, ^∗∗∗^p < 0.001, ^∗∗∗∗^p < 0.0001. All error bars represent the SEM. See also Figures S1–S3.

To approach this issue from a complementary angle, we analyzed the 24-h sleep-wake cycle in *LPO*-Δ*Gal* mice (Figures 1 and S2). Ablation of LPO galanin neurons modestly reduced total wake time and increased total NREM time. These effects were specific for “lights off,” the most active phase of the mice. There was no change in amounts of WAKE/NREM during “lights on” (Figure 1A). REM sleep was unaffected in either lights on or lights off. Furthermore, there were no differences in electroencephalogram (EEG) power between *LPO-Gal-GFP* and *LPO-*Δ*Gal* mice in either the WAKE or NREM states (Figure S2B). Sleep architecture, however, was highly fragmented in *LPO*-Δ*Gal* mice (Figure 1B). The number of WAKE and NREM episodes increased markedly, whereas their durations shortened. These effects were most marked during the lights off period. REM sleep episodes and their durations were not affected (Figure 1B). WAKE-to-NREM and NREM-to-WAKE transitions were significantly increased (Figure 1C), but transitions between other vigilance states did not change. Thus, even allowing for the high sleep-wake fragmentation that appears in *LPO-*Δ*Gal* mice, LPO galanin neurons are dispensable for achieving NREM sleep. Nevertheless, acute chemogenetic activation of LPO^Gal^ neurons with clozapine-*N*-oxide (CNO) in *LPO-Gal-hM*_*3*_*D*_*q*_ mice induced NREM sleep (Figures S3A–S3C), in agreement with earlier reports [21]. The delta power of this CNO-induced NREM sleep in *LPO-Gal-hM*_*3*_*D*_*q*_ mice was higher than the power of NREM sleep after saline injection (Figure S3D). CNO had no effect on sleep in control mice (Figure S3E). Thus, galanin neurons can induce NREM sleep acutely (as first determined in [21]), but in the galanin-lesioned mice, it seems likely that other types of sleep-promoting neurons in the PO and elsewhere still induce sleep [22, 30]; however, galanin neurons are essential for consolidated sleep. Consistently, numbers of galanin neurons in the post-mortem human PO hypothalamus inversely correlate with the degree of sleep-wake fragmentation [33].

### PO Galanin Neurons Chronically Lower Body Temperature

**Figure 2:**
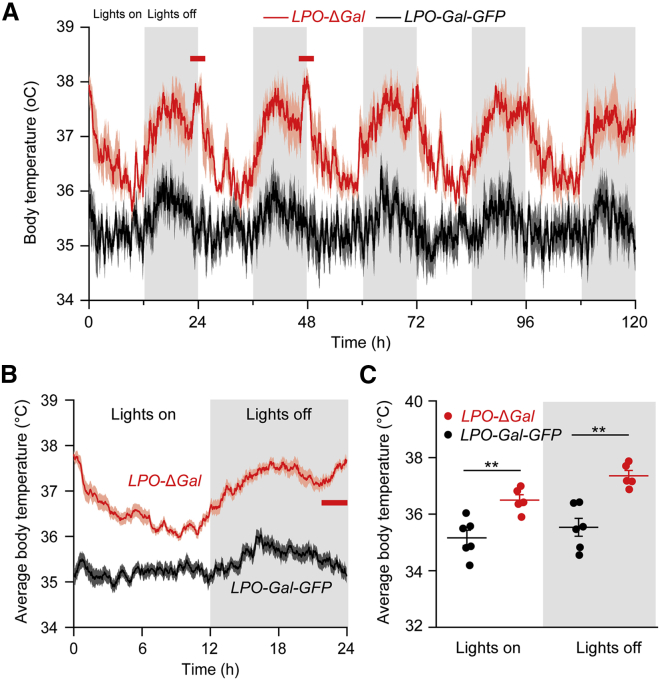
Chronic Ablation of LPO Galanin Neurons Markedly Elevated Core Body Temperature (A) Ablation of LPO^Gal^ neurons caused increases in both the average core body temperature and its diurnal variation. The record shows typical recordings over 5 days for both *LPO-*Δ*Gal* mice (red) and control *LPO-Gal-GFP* mice (black). (B) Average core body temperature over 24 h (*LPO-*Δ*Gal* mice and control *LPO-Gal-GFP* mice) also shows an abrupt and transient increase in body temperature around the transition from lights off to lights on in the *LPO-*Δ*Gal* mice but not the control *LPO-Gal-GFP* mice. (C) Average core body temperature increased in *LPO-*Δ*Gal* mice in both lights on and lights off (^∗∗^p < 0.01, unpaired two-tailed t test) (*LPO-Gal-GFP*, n = 6; *LPO-*Δ*Gal*, n = 5). All error bars represent the SEM. See also Figure S2C, showing that locomotion was unaffected. See also Figures S2 and S4.

Certain PO neurons (e.g., GABA/galanin-, glutamate/NOS1-, PACAP/BDNF-, and TRPM2-expressing cells) respond to immediate external or internal thermal challenge by acutely initiating body cooling or heating [21, 22, 34, 35, 36, 37, 38, 39], but it is unclear whether these cells are chronically regulating body temperature. Five weeks after ablation, *LPO*-Δ*Gal* mice had a striking increase in their core body temperatures compared with *LPO-Gal-GFP* mice (Figures 2A and 2B). In a continuous 5-day recording of body temperature, the mice retained a diurnal variation of body temperature, with higher and lower temperatures during the lights off (active phase) and lights on (inactive phase) periods, respectively (Figures 2A and 2B). However, the average body temperature of *LPO*-Δ*Gal* mice was raised to 37°C, compared with the average 35.5°C of *LPO-Gal-GFP* controls (Figure 2C). The raised body temperature of *LPO*-Δ*Gal* mice was not explained by raised motor activity: *LPO-*Δ*Gal* mice did not have changed locomotor activity in the open-field test (Figures S2C and S2D).

The diurnal range of body temperature change of *LPO*-Δ*Gal* mice increased, reminiscent of the increase in amplitude of body temperature diurnal variation in PO-lesioned rats [25]. In the *LPO-Gal-GFP* control group, the average body temperature during the day and night was around 36°C and 35°C, respectively, whereas the *LPO*-Δ*Gal* group had their average day and night body temperatures around 38°C and 36°C, respectively (Figure 2C). Thus, PO^Gal^ neurons act chronically to lower body temperature. This is supported by chemogenetic experiments, as first reported by others [21]. Indeed, we also found that chemogenetic activation of PO^Gal^ neurons with CNO in *LPO-Gal-hM*_*3*_*D*_*q*_ mice induced hypothermia (Figures S3A, S4B, and S4C). PO galanin neurons are likely to initiate and maintain body cooling via the dorsomedial hypothalamus, rostral raphe pallidus, and rostroventral lateral medulla [38] pathways, which stop brown fat thermogenesis and induce blood vessel dilation.

A new feature emerged in the diurnal core body temperature variation in *LPO-*Δ*Gal* mice: a pronounced positive spike appeared in their body temperature around the transition from lights off to lights on at zeitgeber time (ZT)24 (see red bars in Figures 2A and 2B), suggesting that LPO galanin neurons would normally lower body temperature at the point in the diurnal cycle where sleep pressure is highest at the start of the lights on period. Given that sleep induction occurs when the rate of core body temperature decline is maximal [40, 41], this could explain why *LPO-*Δ*Gal* mice are actually more awake compared with *LPO-Gal-GFP* mice at the start of lights on (Figure 1A).

Although NREM sleep coincides with body cooling, a common perception is that during fever, when we are hot, we sleep more. The circuitry producing sleepiness in fever likely involves the PO [42]. Because *LPO-*Δ*Gal* mice have higher temperatures, a hyperthermia-induced sleep might explain their total NREM sleep being increased from control mice, when in fact we might have expected total sleep time in *LPO-*Δ*Gal* mice to decrease (as a result of losing sleep-promoting galanin neurons). A minimal conclusion, however, is that the NREM sleep occurring during hyperthermia does not require PO galanin neurons. But is hyperthermia, in any case, NREM sleep promoting? In one study, administration to mice of fever-promoting agents (e.g., TNFα and interleukin 1β) caused 12 h of hypothermia with increased amounts of NREM sleep, followed by 12 h of hyperthermia with normal amounts of NREM sleep (see Figure S1 in [43]). Furthermore, in rats, an increase of core body temperature from 37°C to 38°C correlated with decreased sleep time [44]. Finally, in our experiments, during the lights on phase, when sleep drive is highest, *LPO-*Δ*Gal* mice have raised core body temperatures (Figure 2C) but have similar amounts of NREM sleep as control *LPO-Gal-GFP* mice (Figure 1A). Collectively, these examples show that hyperthermia per se does not necessarily increase NREM sleep.

### PO Galanin Neurons Contribute to Sleep Homeostasis

**Figure 3:**
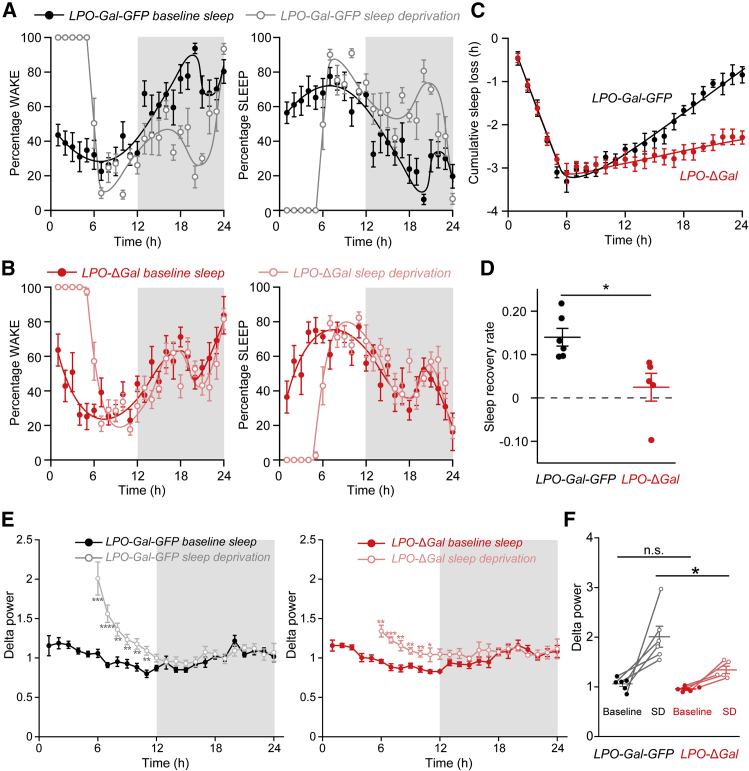
Homeostatic Sleep Rebound Following Sleep Deprivation Was Largely Abolished by the Ablation of LPO Galanin Neurons (A) In control *LPO-Gal-GFP* mice, there was a strong reduction in WAKE and an increase in total sleep (NREM + REM) following 5 h of sleep deprivation. The main effect, compared to the diurnal variation in WAKE and sleep times, occurred during the lights off period following sleep deprivation (which was carried out during the lights on period) (n = 6). (B) In *LPO-*Δ*Gal* mice, however, there was no change in WAKE or total sleep (NREM + REM) time following 5 h of sleep deprivation (n = 5). (C) Most (∼80%) of the sleep lost as a result of 5 h of sleep deprivation was recovered after 19 h in *LPO-Gal-GFP* mice (black; n = 6), whereas only ∼22% of the sleep loss was recovered in *LPO-*Δ*Gal* mice (red; n = 5). (D) The sleep recovery rate after sleep deprivation is significantly reduced in *LPO-*Δ*Gal* mice (red; n = 5) compared with *LPO-Gal-GFP* (black; n = 6) (^∗^p < 0.05, unpaired two-tailed t test). (E) Time course of the delta power (0.5–4 Hz) after 5 h of sleep deprivation in *LPO-Gal-GFP* and *LPO-*Δ*Gal* mice. In *LPO-Gal-GFP*, there was a large increase in delta power after sleep deprivation that decayed over 6 h back to baseline. By contrast, there was a lower increase in delta power after sleep deprivation in *LPO-*Δ*Gal* mice (^∗^p < 0.05, ^∗∗^p < 0.01, ^∗∗∗^p < 0.001, ^∗∗∗∗^p < 0.0001, repeated-measures one-way ANOVA with Holm-Bonferroni post-hoc test). (F) Delta power (0.5–4 Hz) in the first hour of recovery sleep compared with baseline sleep in ZT6 for both *LPO-Gal-GFP* and *LPO-*Δ*Gal* mice. *LPO-Gal-GFP* and *LPO*-Δ*Gal* mice had similar delta power in their baseline sleep but, after 5 h of sleep deprivation, *LPO-Gal-GFP* mice had a larger increase in delta power compared with *LPO-*Δ*Gal* mice (^∗^p < 0.05, unpaired two-tailed t test) (*LPO-Gal-GFP*, n = 6; *LPO-*Δ*Gal*, n = 5). n.s., not significant. All error bars represent the SEM.

The sleep following sleep deprivation is longer and deeper, with an increased power of delta (0.5–4 Hz) oscillations, a phenomenon termed sleep homeostasis [1, 2, 3, 4]. At the circuit level, sleep homeostasis is partly regulated in the PO area [15, 45, 46], as well as locally in the neocortex [47, 48, 49]. To examine how LPO^Gal^ neurons regulate sleep homeostasis, a 5-h sleep deprivation was applied to both groups of mice. In control *LPO-Gal-GFP* mice, there was a reduction in wakefulness and an increase in total sleep (NREM + REM sleep) following 5 h of sleep deprivation (Figure 3A). The main effect, compared to the diurnal variation in wake and sleep times, occurred during the lights off period following sleep deprivation (which was carried out during the lights on period) (Figure 3A). In *LPO-*Δ*Gal* mice, however, there was no change in WAKE or total sleep (NREM + REM) time following 5 h of sleep deprivation (Figure 3B). Most (∼80%) of the sleep lost as a result of 5 h of sleep deprivation was recovered after 19 h in *LPO-Gal-GFP* mice, whereas only ∼22% of the sleep loss was recovered in *LPO-*Δ*Gal* mice (Figures 3C and 3D). Indeed, the sleep recovery rate after sleep deprivation was significantly reduced in *LPO-*Δ*Gal* mice compared with *LPO-Gal-GFP* (Figures 3C and 3D).

We also looked at delta power after sleep deprivation. The delta power of baseline NREM sleep varies characteristically over 24 h (see mouse data in [50]). This delta power baseline was similar between non-sleep-deprived *LPO-Gal-GFP* and *LPO-*Δ*Gal* mice (Figure 3E). After 5-h sleep deprivation, during the first part of the sleep rebound of *LPO-Gal-GFP* mice, the power in the delta wave band was, as expected, increased compared to the delta power during sleep at the equivalent ZT (Figures 3E and 3F) (see the equivalent data in [50]). For the sleep-deprived *LPO-Gal-GFP* mice, the initial sharply increased delta power declined over the first 6 h post-sleep deprivation, until by the start of lights off it was approximately the same as baseline (Figure 3E). The increase in NREM delta power after sleep deprivation, although still significantly above the delta power baseline, was significantly reduced in *LPO-*Δ*Gal* compared with *LPO-Gal-GFP* mice (Figures 3E and 3F). Our results suggest that sleep homeostasis can be regulated globally, via the hypothalamus. Another interpretation is that the sleep homeostasis of *LPO-*Δ*Gal* mice is intact but the capacity of entering deep consolidated sleep with high delta power is deficient, for example as a result of elevated arousal-promoting drive; but because *LPO-*Δ*Gal* mice are actually sleeping slightly more during their active lights off period (Figure 1A), this tends to suggest that their arousal-promoting drive is, if anything, diminished.

### α2-Adrenergic Agonist-Induced Sedation and Hypothermia Require PO Galanin Neurons

**Figure 4:**
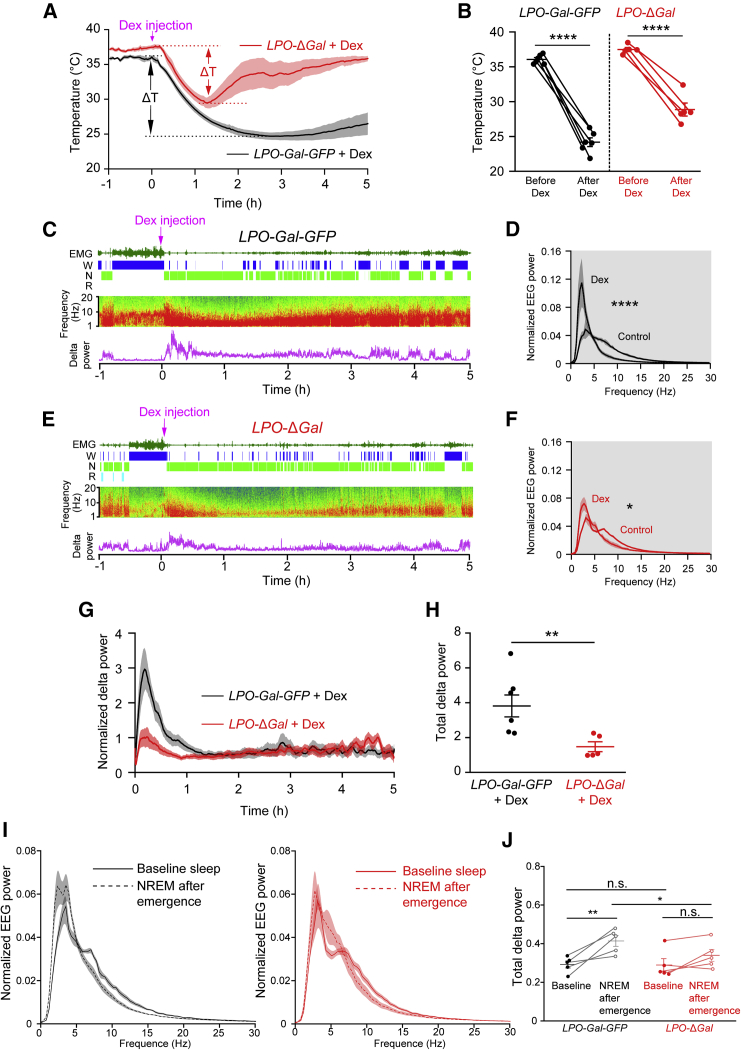
The Characteristic Sustained Hypothermia and Increased NREM-like Delta Power Induced by Dexmedetomidine Were Both Largely Abolished by the Ablation of LPO Galanin Neurons (A) Dexmedetomidine was delivered at ZT19 (lights off), when mice were in their most active phase. In control *LPO-Gal-GFP* mice, there was a strong reduction in temperature from about 36°C to approximately 25°C over the course of 75 min (post-dexmedetomidine induction). This hypothermia persisted beyond 4 h post-injection. In *LPO-*Δ*Gal* mice, however, the initial reduction in body temperature commenced after dexmedetomidine injection lasted only for the first hour and did not reach the same nadir as in *LPO-Gal-GFP* control mice, and the body temperature almost returned to starting levels (33°C ± 1.5°C) over the next hour. (B) The body temperature before, and lowest temperature after, dexmedetomidine injection in *LPO-Gal-GFP* (∗∗∗∗p < 0.0001, paired two-tailed t test; n = 6) and *LPO-*Δ*Gal* mice (∗∗∗∗p < 0.0001, paired two-tailed t test; n = 5). (C) Dexmedetomidine injection of *LPO-Gal-GFP* mice: examples of EEG and EMG raw data and vigilance-state scoring. (D) EEG power spectra of *LPO-Gal-GFP* mice, averaged over 30 min after dexmedetomidine injections. Dexmedetomidine induced a large increase in delta power relative to that of natural NREM sleep (control spectrum) (^∗∗∗∗^p < 0.0001, paired two-tailed t test; n = 6). (E) Dexmedetomidine injection into *LPO-*Δ*Gal* mice: examples of EEG and EMG raw data and vigilance-state scoring. (F) EEG power spectra of *LPO-*Δ*Gal* mice, averaged over 30 min after dexmedetomidine injection. Dexmedetomidine induced a small increase in delta power relative to that of natural NREM sleep (control spectrum) (∗p < 0.05, paired two-tailed t test; n = 5). (G) Time courses of evoked NREM-like delta power following dexmedetomidine i.p. administration at ZT19 to *LPO-Gal-GFP* (black; n = 6) and *LPO-*Δ*Gal* mice (red; n = 5). (H) In *LPO-Gal-GFP* control mice (black; n = 6), within 20 min of injection, dexmedetomidine induced a large increase in delta power relative to that of NREM sleep, but this was substantially less in *LPO-*Δ*Gal* mice (red; n = 5) (^∗∗^p < 0.01, unpaired two-tailed t test). (I) 3.5 h after *LPO-Gal-GFP* mice (black; n = 5) were given 50 μg/kg dexmedetomidine, the delta power band in NREM sleep was elevated compared with that of baseline NREM sleep at the corresponding circadian time. This effect was not present in *LPO-*Δ*Gal* mice (red; n = 5). (J) Quantification of delta rebound effect 3.5 h after dexmedetomidine in *LPO-Gal-GFP* mice and its absence in *LPO-DGal* mice (∗p < 0.05, ∗∗p < 0.01; two-way ANOVA and Holm-Bonferroni post hoc test). All error bars represent the SEM.

Hypothermia is an unwanted side effect of dexmedetomidine [15, 19]. Indeed, in control *LPO-Gal-GFP* mice, after injection (intraperitoneal; i.p.) 50 μg/kg dexmedetomidine, there was a strong reduction in core body temperature from about 36°C to 25°C over the course of 2 h (post-dexmedetomidine injection) (Figures 4A and 4B). This hypothermia persisted beyond 4 h post-injection. In *LPO-*Δ*Gal* mice, however, the initial reduction in body temperature after dexmedetomidine injection lasted only for the first hour and did not reach the same nadir as in *LPO-Gal-GFP* control mice, and the body temperature returned to normal levels over the next hour (Figures 4A and 4B). That the initial phase of body cooling triggered by dexmedetomidine still happens in the *LPO-*Δ*Gal* mice could be because α2A receptors are found on smooth muscle of peripheral blood vessels and so dexmedetomidine will promote heating loss directly by vasodilation of tail veins.

We investigated whether ablating LPO^Gal^ neurons compromised dexmedetomidine’s ability to induce an NREM-like sleep state (Figures 4C–4F). After 1 h of EEG and temperature recordings, animals received 50 μg/kg dexmedetomidine (i.p.) in the lights off period, their active phase. In *LPO-Gal-GFP* control mice, within 20 min of injection, dexmedetomidine induced a large increase in delta power relative to that in NREM sleep occurring at the same ZT (Figures 4C and 4D), but this increase was substantially weaker in *LPO-*Δ*Gal* mice (Figures 4E and 4F). (Note: REM sleep was nearly abolished after dexmedetomidine administration to both *LPO-Gal-GFP* and *LPO-*Δ*Gal* groups.) Looking at the time course of the evoked delta power following dexmedetomidine injection (Figures 4G and 4H), in *LPO-Gal-GFP* mice the delta power peaked at 11 min post-injection and then declined over the following hours even though the mice were still in an NREM-like sleep. (Because of the evolving hypothermia in the dexmedetomidine-injected *LPO-Gal-GFP* mice [Figure 4A], the power of the EEG spectrum declines with time as we and others documented previously; however, the vigilance state can still be scored as NREM sleep-like [22, 51].) By contrast, the delta power remained at normal NREM sleep levels in dexmedetomidine-injected *LPO*-Δ*Gal* mice, even at the start of the experiment (Figures 4G and 4H). (If wild-type animals that are given dexmedetomidine are kept warm, the high NREM-like delta power that is triggered by the drug does not diminish [unpublished data].)

Because daily torpor in some species leads to a rebound of delta power during subsequent NREM sleep [52, 53], we asked by analogy whether dexmedetomidine also caused a delta power rebound. Indeed, looking at NREM sleep 3.5 h after dexmedetomidine injection, there was a rebound in delta power when baseline NREM sleep resumed after dexmedetomidine injection. The rebound was absent in *LPO-*Δ*Gal* mice (Figures 4I and 4J), highlighting a further link between the mechanism of dexmedetomidine-induced sedation and the sleep homeostasis machinery.

The metabotropic α2A-adrenergic receptor mediates both the NREM sleep-like state and the hypothermic effects of dexmedetomidine [54, 55]. Although it is commonly thought that dexmedetomidine induces sedation by inhibiting noradrenaline release from neurons in the locus ceruleus [55, 56, 57], there is evidence that this is not the case [15, 18, 58]: VLPO lesions in rats blunt the ability of dexmedetomidine to induce sedation [16]; dexmedetomidine induces cFOS expression in the PO [15, 16], and can induce sedation even when noradrenaline release from the locus ceruleus is genetically removed [58]; and reactivating ensembles of PO neuron activity tagged during dexmedetomidine-induced is sufficient to induce NREM sleep and hypothermia [15]. Dexmedetomidine is expected to directly excite PO neurons by influencing hyperpolarization-activated cyclic nucleotide-gated cation channels [59].

### Subtypes of PO Galanin Neurons

In the simplest case, one PO galanin neuron type, sensitive to the factors driving sleep homeostasis and having α2A receptors, induces and maintains NREM sleep and body cooling. This seems unlikely. In fact, single-cell profiling and multiplex *in situ* labeling found multiple subtypes of galanin-expressing neuron, dispersed and intermingled, in the mouse PO region [28]. Most of these galanin neurons are GABAergic, but several are glutamatergic, and one *vgat*/galanin subtype expresses tyrosine hydroxylase and the vesicular monoamine transporter [28]. To untangle the circuit logic, intersectional genetics combined with c-FOS-based activity tagging could be deployed [22].

### Conclusions

PO galanin neurons are required for consolidated NREM sleep, and mediate both rebound NREM sleep linked to body cooling and adrenergic agonist-induced sedation with sustained hypothermia. PO galanin neuronal activation causes higher delta power (i.e., a more synchronized state of neocortical activity), whether through sleep homeostasis following sleep deprivation or chemogenetic and dexmedetomidine activation. We and others have suggested that strengthening this sleep and cooling process, triggered through, e.g., PO glutamate/NOS1 neurons and galanin neurons, could cause torpor [21, 22]. It seems likely that dexmedetomidine, through galanin neurons, overactivates the natural sleep homeostasis pathway, pushing the animal into deep hypothermia, from which there is a subsequent delta NREM rebound. This suggests that the dexmedetomidine-induced sedation is not restorative like normal sleep, possibly because the mice become too cold. Identifying the cellular substrates of dexmedetomidine’s sedative actions could help discover drugs causing strong sedation without excessive cooling.

## STAR★Methods

### Key Resources Table

**Table:** 

REAGENT or RESOURCE	SOURCE	IDENTIFIER
**Antibodies**		

Anti-EGFP rabbit polyclonal antibody	Thermo Fisher Scientific	A6455
Anti-mCherry mouse monoclonal antibody	Clontech	632543; RRID: AB_2307319
Rabbit polyclonal anti-parvalbumin	Abcam	ab11427
Alexa Flour 488 goat anti-rabbit IgG	Molecular Probes	A11034; RRID: AB_2576217
Alexa Flour 594 goat anti-mouse IgG	Molecular Probes	A11005; RRID: AB_141372

**Chemicals, Peptides, and Recombinant Proteins**		

Isoflurane	Zoetis	50019100
Clozapine *N*-Oxide	Tocris	4936
Dexmedetomidine	Tocris	2749

**Experimental Models: Cell Lines**		

HEK293 cells	Sigma-Aldrich	85120602/CVCL_0045

**Experimental Models: Organisms/Strains**		

Mouse: Tg(Gal-cre)KI87Gsat/Mmucd	Mutant Mouse Regional Resource Center	stock No. 031060-UCD

**Oligonucleotides**		

Galanin primers	Mutant Mouse Regional Resource Center	Primers as recommended

**Recombinant DNA**		

Adenovirus helper plasmid *pFΔ6*	Donated by M Klugmann	N/A
AAV helper plasmid *pH21* (AAV1)	Donated by M Klugmann	N/A
AAV helper plasmid pRVI (AAV2)	Donated by M Klugmann	N/A
pAAV-EF1α-flex-taCasp3-TEVp	Donated by Nirao Shah	Addgene #45580
pAAV-CAG-flex-GFP	Donated by Edward Boyden	Addgene #28304
pAAV-hSyn-flex-hM3Dq-mCherry	Donated by Bryan Roth	Addgene #44361

**Software and Algorithms**		

Spike2 v7.18	Cambridge Electronic Design	http://ced.co.uk/products/spkovin
MATLAB (Version R2016b)	MathWorks	https://uk.mathworks.com/
Downloader (Version 1.27)	Evolocus	http://www.evolocus.com
ImageJ	Fiji	https://fiji.sc/
Activity Monitor Version 5 for mice	Medical Associates	http://www.med-associates.com/product-category/activity-software/

**Other**		

1-ml HiTrap Heparin column	Sigma-Aldrich	5-4836
Angle Two stereotaxic frame	Leica Microsystems	N/A
Hamilton microliter 10-μl syringes	Hamilton	701
Neurologger 2A	Alexei L. Vyssotski	N/A
Temperature logger	STAR-ODDI	DST nano-T

### Lead Contact and Materials Availability

Further information and requests for resources and reagents should be directed to, and will be fulfilled by, the Lead Contact, William Wisden (w.wisden@imperial.ac.uk). Please note that this study did not generate new unique reagents.

### Experimental Model and Subject Details

#### Mice

Animal care and experiments were performed under the UK Home Office Animal Procedures Act (1986) and were approved by the Imperial College Ethical Review Committee. *Gal-Cre* mice (Tg(Gal-cre)KI87Gsat/Mmucd) were generated by GENSAT and deposited at the Mutant Mouse Regional Resource Center, stock No. 031060-UCD, The Gene Expression Nervous System Atlas (GENSAT) Project (NINDS Contracts N01NS02331 & HHSN271200723701C to The Rockefeller University, New York) [26]. In this mouse line, Cre recombinase expression is driven from a bacterial artificial chromosome transgene containing the endogenous mouse galanin gene. All mice used in the experiment were equally mixed genders and had the first surgery at the age of 10-12 weeks. Mice were housed individually. *Ad libitum* food and water were available for all mice and a reversed 12 h:12 h light/dark cycle (“lights on” hours: 17:00-05:00) with constant temperature and humidity.

### Method Details

#### AAV transgene plasmids

AAV transgenes had a flexed reading frame in an inverted orientation, and therefore could only be activated by Cre recombinase. The *pAAV-EF1α-flex-taCasp3-TEVp* transgene plasmid was Addgene plasmid #45580 (a gift from Nirao Shah) [60]. The *pAAV-CAG-flex-GFP* transgene construct was Addgene plasmid #28304 (a gift from Edward Boyden). The *pAAV-hSyn-flex-hM*_*3*_*D*_*q*_*-mCherry* transgene construct was Addgene plasmid #44361 (a gift from Bryan Roth) [61].

#### Generation of recombinant AAV particles

All AAV transgenes were packaged in our laboratory into AAV capsids with a mixed serotype 1 & 2 (1:1 ratio of AAV1 and AAV2 capsid proteins) as described previously [62].

#### Surgeries and stereotaxic injection of AAV

For surgery, mice were anesthetized with an initiation concentration of 2.5% isoflurane in O_2_ (vol/vol) by inhalation and mounted into a stereotaxic frame (Angle Two, Leica Microsystems, Milton Keynes, Buckinghamshire, UK). Mice were maintained anesthetized on 2% isoflurane during surgery. A heat pad was used during the whole surgery to prevent heat loss. For ablating galanin neurons, the two AAV viruses, AAV*-EF1α-flex-taCasp3-TEVp* and AAV*-CAG-flex-GFP* were mixed in a 1:1 ratio prior to injection while a single virus type was injected for the rest of experiments unless otherwise stated. For the parvalbumin quantification experiment in Figure S1E, AAV-*flex-mCherry* was injected instead of AAV-*CAG-flex-GFP*. AAV viruses were delivered using a 10 μL syringe (Hamilton microliter, #701) with a 33-gauge stainless steel needle (point style 3, length 1.5 cm, Hamilton). The injection coordinates (bilateral) for the LPO relative to Bregma were: AP +0.02 mm; ML ± 0.75 mm; DV was consecutive starting −5.8 (1/2 volume), −5.6 (1/2 volume). A total volume of 0.2-0.5 μL of virus was injected into each hemisphere depending on the viral titration. Mice were allowed three weeks for recovery in their home cage before fitting with Neurologger 2A devices (see section below) and performing behavioral experiments. For experiments where temperature recordings were necessary, temperature loggers were usually inserted (abdominally) two to three weeks after mice had had their viral injection surgeries.

#### EEG and EMG recordings and vigilance states scoring

Non-tethered EEG and EMG recordings were captured using Neurologger 2A devices [63]. Screw electrodes were chronically inserted into the skull of mice to measure cortical EEG using the following coordinates: −1.5 mm Bregma, + 1.5 mm midline - first recording electrode; + 1.5 mm Bregma, −1.5 mm midline – second recording electrode; −1 mm Lambda, 0 mm midline – reference electrode. EMG signals were recorded by a pair of stainless steel electrodes implanted in the dorsal neck muscle. Four data channels (2 of EEG and 2 of EMG) were recorded with four times oversampling at a sampling rate of 200 Hz. The dataset was downloaded and waveforms visualized using Spike2 software (Cambridge Electronic Design, Cambridge, UK) or MATLAB (MathWorks, Cambridge, UK). The EEG signals were high-pass filtered (0.5 Hz, −3dB, an FFT size of 512 was the designated time window) using a digital filter and the EMG was band-pass filtered between 5-45 Hz (−3dB). Power in the delta (0.5-4 Hz), theta (6-10 Hz) bands and theta to delta band ratio were calculated, along with the root mean square (RMS) value of the EMG signal (averaged over a bin size of 5 s). All of these data were used to define the vigilance states of WAKE, NREM and REM by an automatic script. Each vigilance state was screened and confirmed manually afterward. The peak frequency during NREM epochs were analyzed using Fourier transform power spectra to average power spectra over blocks of time.

#### Core body temperature recording

Core body temperature was recorded using temperature loggers (DST nano, Star-Oddi, HerfØlge, Denmark) implanted abdominally. A pre-defined program was set to sample the temperature data every two minutes for baseline core body temperature and drug/vehicle administration. At the end of the experiments, the loggers were retrieved and the data were downloaded and analyzed.

#### Sleep deprivation and recovery sleep

The sleep deprivation protocol was similar to the one we used before [15], and started at ZT zero (17:00), the start of the “lights on” period when the sleep drive of the mice is at its maximum. Both experimental and control groups were removed from their home cages and sleep deprived for 5 hours by introducing novel objects or gently tapping on the cages. Handling was kept to a minimum. After sleep deprivation, mice were immediately placed back in their home cages. EEG and EMG signals were recorded during the sleep deprivation and recovery periods.

#### Chemogenetics and behavioral assessment

For chemogenetic activation, clozapine-N-oxide (CNO) (C0832, Sigma-Aldrich) was used. 1 mg/kg of CNO dissolved in saline or saline in the same volume was administrated by intraperitoneal (*i.p.*) injection and the EEG/EMG data and core body temperature were recorded. Mice were split into random groups that either received CNO or saline injection for an unambiguous comparison. Drugs were administrated at ZT18 (11:00, “lights off”) when the mice were in their most active period and had their highest body temperature.

#### Dexmedetomidine experiments

Prior to dexmedetomidine injection, animals with implanted temperature loggers were fitted with Neurologger 2A devices, and one hour of both baseline EEG/EMG data and core body temperature were recorded as reference. Dexmedetomidine (Tocris Bioscience) was dissolved in saline to make a final concentration of 50 μg per kg and delivered *i.p.* at ZT19 (12:00, “lights-off”). Animals were placed back to their home cage immediately after injection for a further five-hour recording and the EEG/EMG data and core body temperature were simultaneously recorded. A six-hour baseline recording from the same mouse of its natural sleep-wake cycle and core body temperature between ZT18 to ZT24 (11:00-17:00, “lights off”) were used for parallel comparison with the dexmedetomidine injection experiments.

#### Immunohistochemistry

Mice were fixed by transcardial perfusion with 4% paraformaldehyde (Thermo scientific) in PBS, pH 7.4 after deep anesthesia by pentobarbital (100 mg/kg body weight; *i.p.*). Brains were removed and preserved in 30% sucrose in PBS. 35 μm-thick coronal sections were sliced using a Leica VT1000S vibratome. Free-floating sections were washed three times in PBS each for 5 minutes, permeabilized in 0.4% Triton X-100 in PBS for 30 minutes, blocked by incubation with 5% normal goat serum (NGS) (Vector) plus 0.2% Triton X-100 in PBS for 1 hour (all performed in room temperature) and then incubated with antisera for GFP (rabbit, 1:1000, Life Technology, #A6455), mCherry (mouse, 1:1000, Clontech, #632543), or parvalbumin (rabbit, 1:1000, Abcam, #ab11427). Primary antisera were diluted in PBS with 2% NGS overnight at 4°C. The following day, primary antisera incubated sections were washed three times in PBS each for 10 minutes and subsequently incubated for 2 hours at room temperature in PBS with 2% NGS plus a dilution of an Alexa Fluor 488 goat anti-rabbit IgG (H+L) (1:1000, Molecular Probes, #A11034) or Alexa Fluor 594 goat anti-mouse IgG (H+L) (1:1000, Molecular Probes, #A11005). Sections were washed 4 times in PBS each for 10 minutes at room temperature and subsequently mounted on glass slides in Vectashield with mounting medium which contains DAPI (H-1200, Vector Laboratories).

### Quantification and Statistical Analysis

Origin v8.6 and Prism6 were used for statistical analyses. Data collection and processing were randomized or performed in a counter-balanced way. The sample sizes and statistical test for each experiment are stated in the figure legends. Data are represented as the mean ± SEM. For the behavioral tests, two-tailed t tests, or one or two-way ANOVA were performed accordingly. p values are shown when they are less than 0.05 (^∗^p < 0.05, ^∗∗^p < 0.01, ^∗∗∗^p < 0.001, ^∗∗∗∗^p < 0.0001). When multiple comparisons were made, the Holm-Bonferroni post hoc test was applied. Mice were excluded from the analysis if the histology did not confirm significant AAV transgene expression in the LPO area of the hypothalamus, or if the transgene expression had spread beyond the target region. Investigator were not blind to treatments.

### Data and Code Availability

This study did not generate any datasets or code.

## Supplemental Information

Document S1. Figures S1–S4

Document S2. Article plus Supplemental Information
