# Supplementary material for: Galanin Neurons Unite Sleep Homeostasis and α2-Adrenergic Sedation
Source: Curr Biol. 2019 Oct 7;29(19):3315–3322.e3. doi: 10.1016/j.cub.2019.07.087 (PMC6868514; doi:10.1016/j.cub.2019.07.087)
Supplement: Document S1. Figures S1–S4 [file mmc1.pdf]

Current Biology, Volume *29*

Supplemental Information

Galanin Neurons Unite Sleep Homeostasis and α2-Adrenergic Sedation

Ying Ma, Giulia Miracca, Xiao Yu, Edward C. Harding, Andawei Miao, Raquel Yustos, Alexei L. Vyssotski, Nicholas P. Franks, and William Wisden

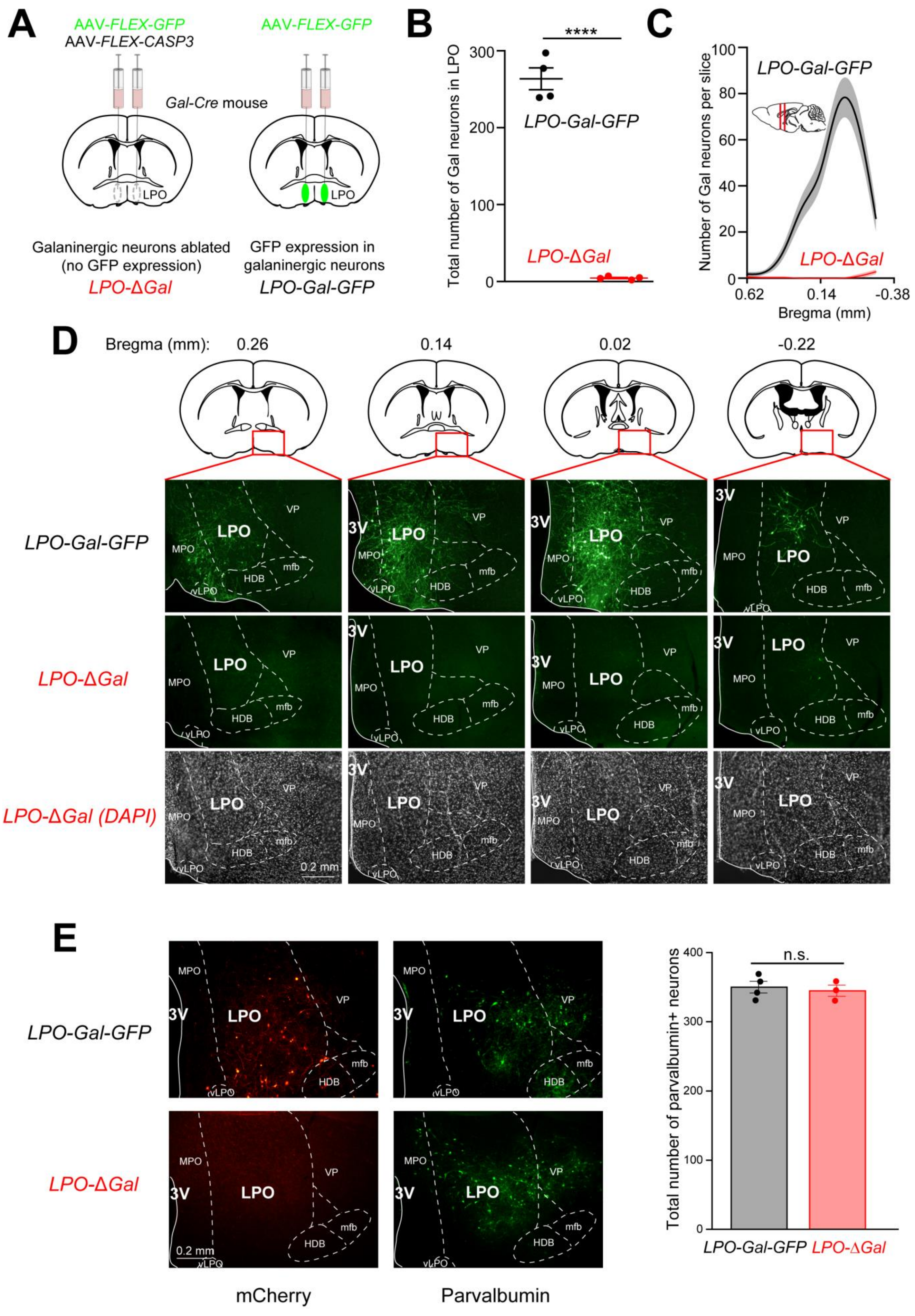
A
AAV-FLEX-GFP
AAV-FLEX-CASP3
AAV-FLEX-GFP
Gal-Cre mouse
LPO
LPO
Galaninergic neurons ablated (no GFP expression)
LPO-ΔGal
GFP expression in galaninergic neurons
LPO-Gal-GFP
B
****
Total number of Gal neurons in LPO
LPO-Gal-GFP
LPO-ΔGal
C
Number of Gal neurons per slice
LPO-Gal-GFP
LPO-ΔGal
Bregma (mm)
D
Bregma (mm): 0.26 0.14 0.02 -0.22
LPO-Gal-GFP
LPO-ΔGal
LPO-ΔGal (DAPI)
LPO
MPO
VP
HDB
mfb
vLPO
3V
0.2 mm
E
LPO-Gal-GFP
LPO-ΔGal
mCherry
Parvalbumin
n.s.
Total number of parvalbumin+ neurons
LPO-Gal-GFP LPO-ΔGal

Figure S1. Ablation of galanin neurons in the LPO of the hypothalamus. Related to Figure 1. (A) Galanin neurons in the LPO of *Gal-Cre* mice were ablated by bilaterally injecting AAV-*FLEX-CASP3*, together with AAV-*FLEX-GFP* to give *LPO-ΔGal* mice. Control animals (*LPO-Gal-GFP* mice) were injected with AAV-*FLEX-GFP* alone. (B) Most (~98%) galanin neurons in the LPO were ablated (****P<0.0001, paired two-tailed t-test). (C) Rostral-caudal distribution of galanin neurons before and after ablation. (D) Example images of galanin neurons in the LPO determined by GFP expression (top row) and after ablation (middle row). The bottom row shows the corresponding DAPI stains from the above immune-stained sections and alignment with the mouse brain atlas (The Allen Brain Atlas, http://www.brain-map.org). (E) Both *LPO-ΔGal* and *LPO-Gal-GFP* sections were stained for parvalbumin expression. Example expression patterns of parvalbumin at Bregma 0.14 were similar in both groups, and further quantification across the whole LPO area demonstrated LPO^{Parv} neurons were not affected by ablation of LPO^{Gal} neurons (unpaired two-tailed t-test). For this particular experiment, AAV-*FLEX-mCherry* was injected to both mouse groups instead of AAV-*FLEX-GFP* (to avoid antibody species cross reactivity). For the data in B, C and E, brain sections containing the LPO area from 4 individual mice were selected in each group, *i.e.* 4 brain sections for each coordinate in one group. 3V, the third ventricle; HDB, magnocellular preoptic nucleus; LPO, lateral preoptic; mfb, medial forebrain bundle; MPO, medial preoptic nucleus; vLPO, ventrolateral preoptic nucleus; VP, ventral pallidum. All error bars represent the SEM.

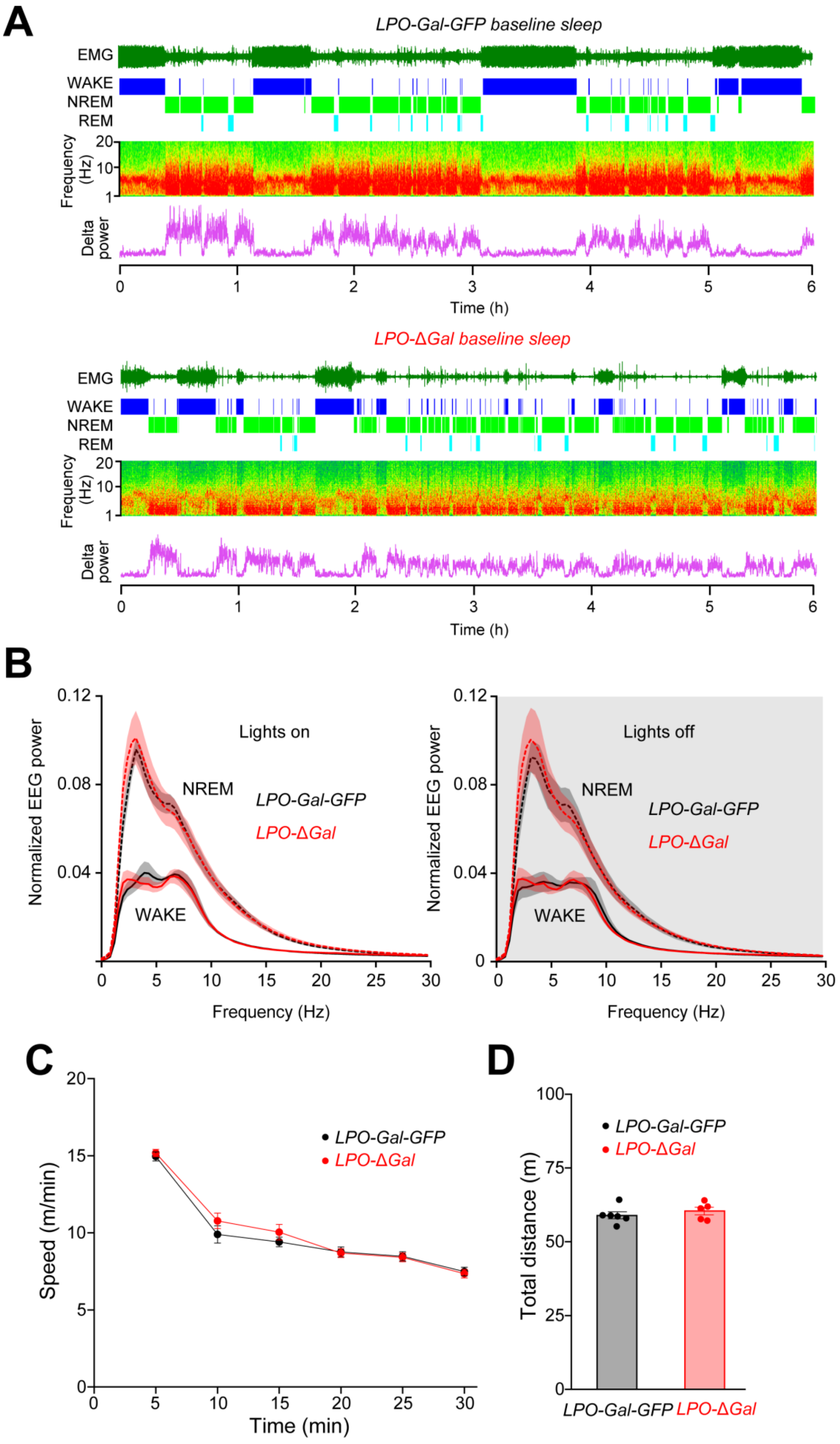
A
LPO-Gal-GFP baseline sleep
EMG
WAKE
NREM
REM
Frequency (Hz)
20
10
1
Delta power
0
1
2
3
4
5
6
Time (h)
LPO-ΔGal baseline sleep
B
Normalized EEG power
0.12
0.08
0.04
0
Lights on
Lights off
NREM
WAKE
LPO-Gal-GFP
LPO-ΔGal
Frequency (Hz)
0
5
10
15
20
25
30
C
Speed (m/min)
Time (min)
D
Total distance (m)
100
75
50
25
0
LPO-Gal-GFP
LPO-ΔGal

Figure S2. Sleep and locomotion in *LPO-Gal-GFP* mice and *LPO-ΔGal* mice. Related to Figure 1 and Figure 2. (A) Examples of EEG and EMG raw data and vigilance-state scoring for *LPO-Gal-GFP* mice and *LPO-ΔGal* mice (from ZT0 to ZT6). (B) EEG power normalized such that the area under the curve was unity during the waking state. There were no significant differences in the EEG power in either the WAKE state or NREM state between *LPO-Gal-GFP* mice and *LPO-ΔGal* mice (*LPO-Gal-GFP*, n=8; *LPO-ΔGal*, n=6). (C, D) The locomotion and speed of *LPO-Gal-GFP* (black; n=6) and *LPO-ΔGal* (red; n=5) mice were determined for 30 mins with an open field test in the "lights off" period (around ZT 18). No differences between the two groups were found (P>0.93, repeated measures one-way ANOVA with post hoc test for C; P>0.98, unpaired two-tailed t-test for D). All error bars represent the SEM.

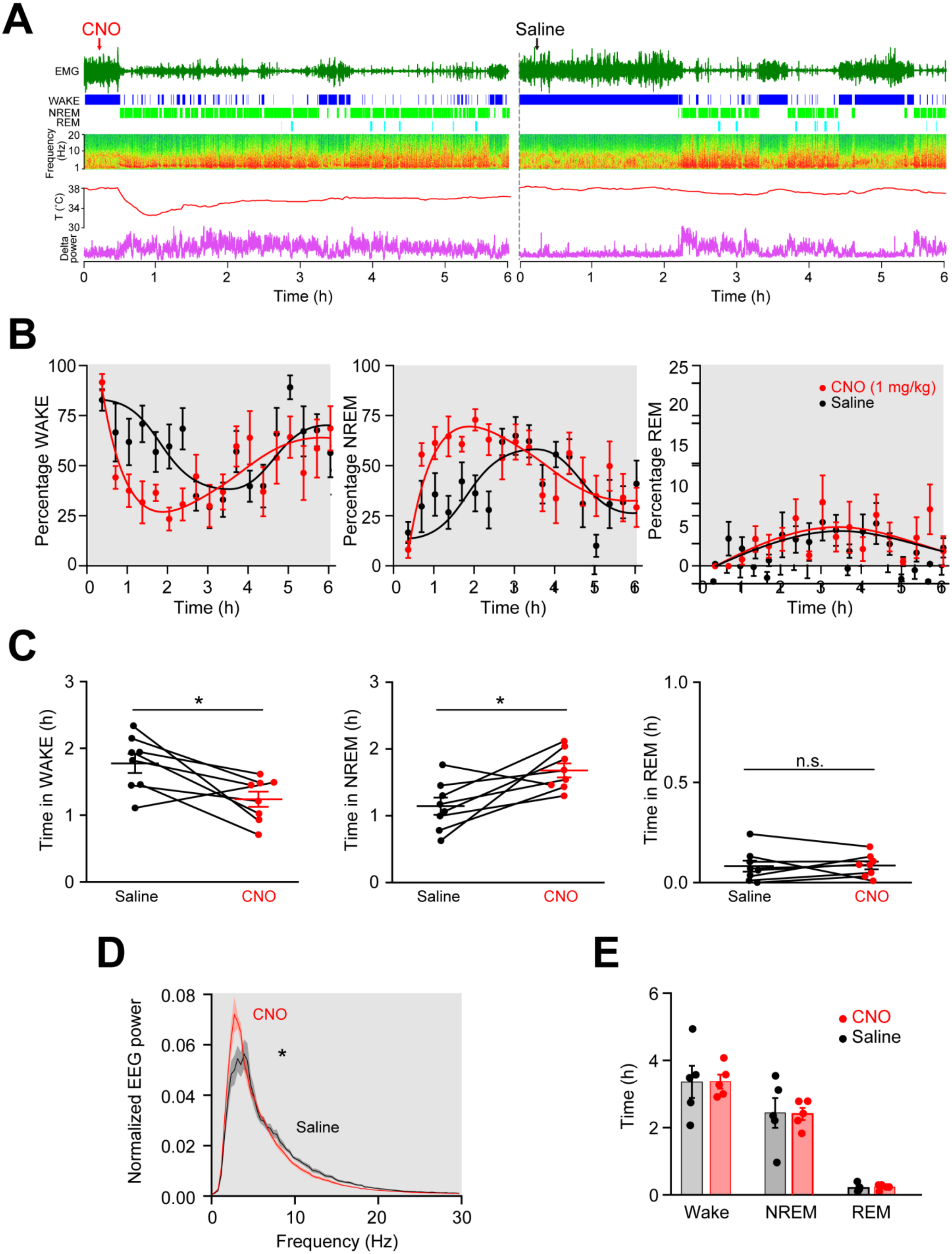

A
CNO
Saline
EMG
WAKE
NREM
REM
Frequency (Hz)
T (°C)
Delta power
Time (h)
B
Percentage WAKE
Percentage NREM
Percentage REM
CNO (1 mg/kg)
Saline
Time (h)
C
Time in WAKE (h)
Time in NREM (h)
Time in REM (h)
n.s.
Saline
CNO
D
Normalized EEG power
CNO
Saline
Frequency (Hz)
E
Time (h)
CNO
Saline
Wake
NREM
REM

Figure S3. Selective chemogenetic activation of LPO^{Gal} neurons by CNO increased NREM sleep. Related to Figure 1. (A) Examples of EEG and EMG raw data and vigilance-state scoring, core body temperature and delta power for *LPO-Gal-*hM_3D_q mice following CNO (1 mg/kg) (left) or saline (right) *i.p.* injection. (B) The percentage of WAKE reduced and the percentage of NREM increased following CNO injection compared with control saline injection. The percentage of time in REM did not change (*n*=8). (C) Total time in WAKE, NREM and REM over the three hours following injection. (**P*<0.05, paired two-tailed *t*-test; *n*=8). (D) The delta power of the CNO-induced NREM-like sleep had a significantly higher power than that of NREM sleep after saline injection (at the same time of injection) (**P*<0.05, paired two-tailed *t*-test; *n*=8). Traces over the first 3 hours post injection. (E) Control for off-target effects: CNO (1 mg/kg) injected *i.p.* into *Gal-Cre* mice (that had not been injected with AAV-*FLEX-*hM_3D_q) did not induce NREM sleep above normally occurring amounts over the next 6 hours (compared with *Gal-Cre* mice that had received saline injections). All error bars represent the SEM.

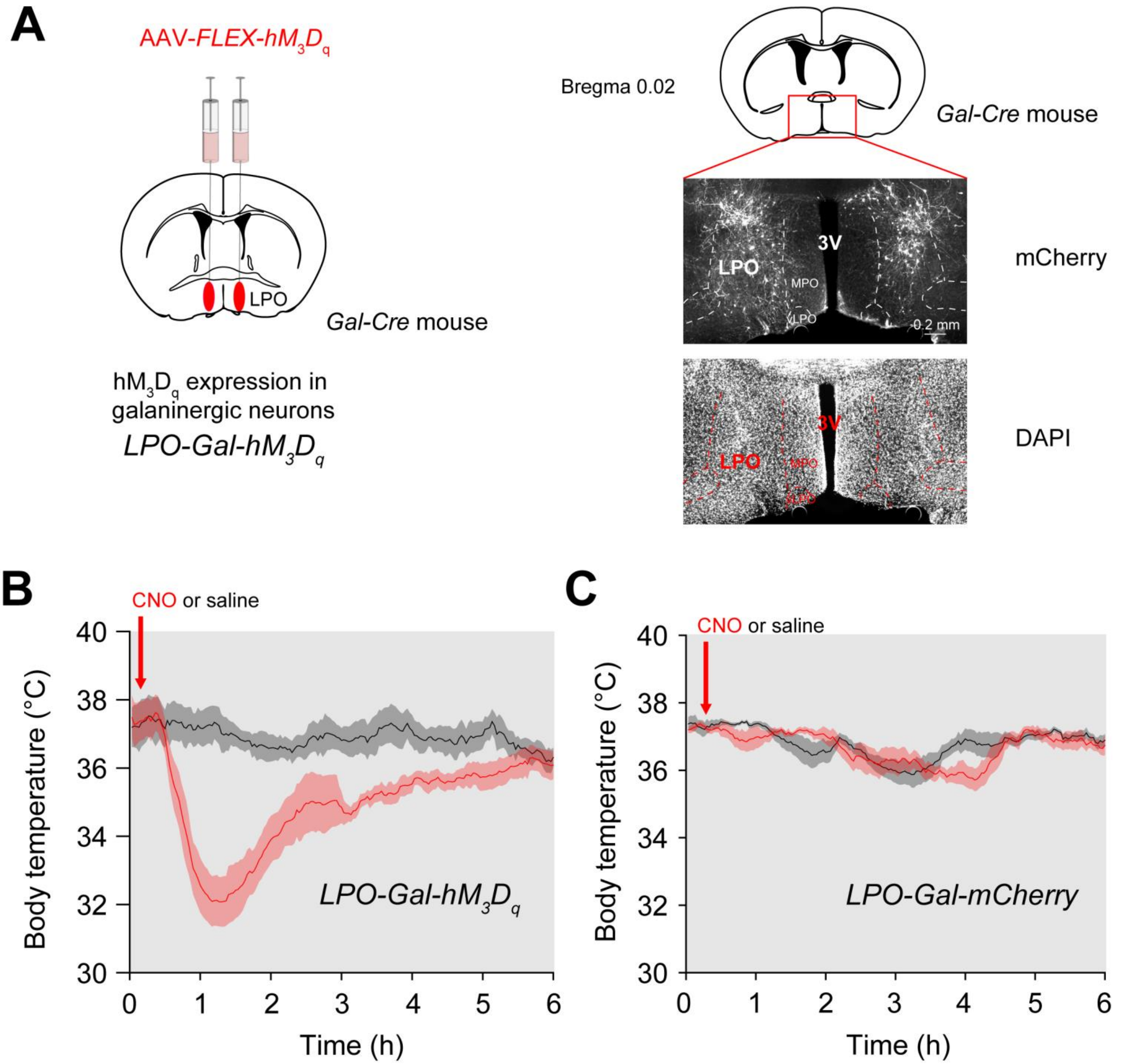
A
AAV-FLEX-hM3Dq
LPO
Gal-Cre mouse
hM3Dq expression in galaninergic neurons
LPO-Gal-hM3Dq
Bregma 0.02
Gal-Cre mouse
3V
LPO
MPO
VLPO
0.2 mm
mCherry
DAPI
B
CNO or saline
Body temperature (°C)
Time (h)
LPO-Gal-hM3Dq
C
CNO or saline
Body temperature (°C)
Time (h)
LPO-Gal-mCherry

Figure S4. Selective chemogenetic activation of LPO^{Gal} neurons induces hypothermia. Related to Figure 2. (A) Galanin neurons in the LPO of *Gal-Cre* mice were made selectively sensitive to CNO by bilaterally injecting AAV-*FLEX-hM$_3$D$_q$* to generate *LPO-Gal-hM$_3$D$_q$* mice. The hM$_3$D$_q$ receptor is fused to the mCherry protein, permitting visualization of receptor expression by immunohistochemistry with mCherry antibodies. The corresponding DAPI staining is shown below. Receptor expression was largely confined to the LPO area. 3V, the third ventricle; LPO, lateral preoptic nucleus; MPO, medial preoptic nucleus; vLPO, ventrolateral preoptic nucleus. (B) CNO (1 mg/kg) but not saline injection (*i.p.*) induced a strong acute hypothermia in *LPO-Gal-hM$_3$D$_q$* mice lasting several hours (*n*=5). (C) Control for off-target effects. CNO (1 mg/kg) injected (*i.p.*) into *Gal-Cre* mice (that had not been injected with AAV-*FLEX-hM$_3$D$_q$*) did not induce hypothermia compared with *Gal-Cre* mice that had received saline injections (*n*=5). All error bars represent the SEM.
