# Supplementary material for: Galanin Neurons Unite Sleep Homeostasis and α2-Adrenergic Sedation
Source: Curr Biol. 2019 Oct 7;29(19):3315–3322.e3. doi: 10.1016/j.cub.2019.07.087 (PMC6868514; doi:10.1016/j.cub.2019.07.087)
Supplement: Document S2. Article plus Supplemental Information [file mmc2.pdf]

Report

Current Biology

Galanin Neurons Unite Sleep Homeostasis and α2-Adrenergic Sedation

Graphical Abstract

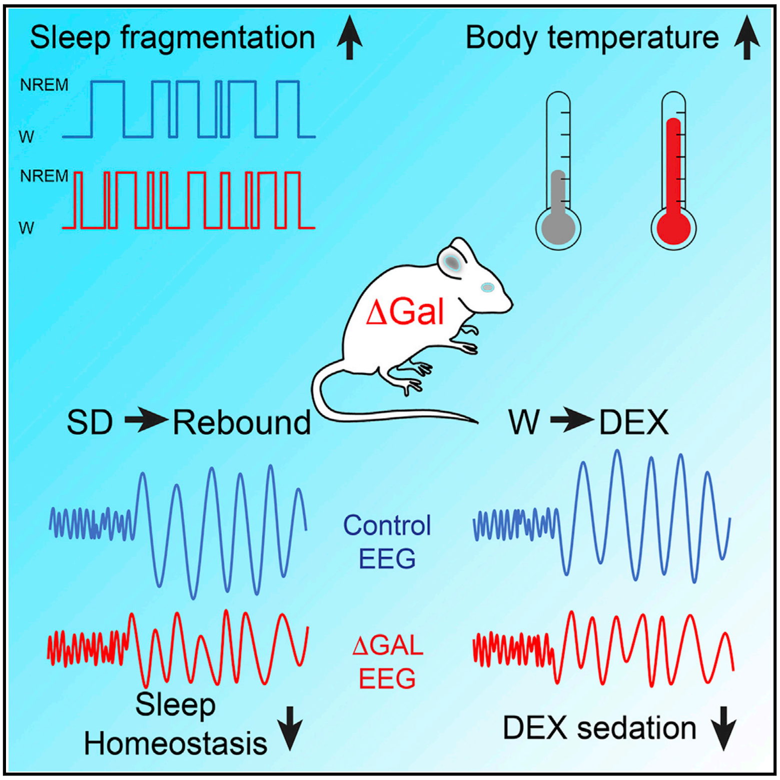

Authors

Ying Ma, Giulia Miracca, Xiao Yu, ..., Alexei L. Vyssotski, Nicholas P. Franks, William Wisden

Correspondence

n.franks@imperial.ac.uk (N.P.F.),
w.wisden@imperial.ac.uk (W.W.)

In Brief

Sleep deprivation increases the urge to sleep. The subsequent sleep rebound is termed sleep homeostasis. Ma et al. find that sleep homeostasis, and its concomitant body cooling, is controlled by preoptic galanin neurons. These same neurons are also needed for α2-adrenergic drugs, such as dexmedetomidine, to induce sedation and cooling.

Highlights

- This is the first identification of a cell type underlying sleep homeostasis
- Preoptic galanin neurons are essential for sleep homeostasis
- Galanin neurons mediate the sedative and hypothermic actions of dexmedetomidine
- Dexmedetomidine causes an EEG delta power rebound dependent on galanin neurons

Ma et al., 2019, Current Biology *29*, 3315–3322
October 7, 2019 © 2019 The Author(s). Published by Elsevier Ltd.
https://doi.org/10.1016/j.cub.2019.07.087

Current Biology
Report

Galanin Neurons Unite Sleep Homeostasis and α2-Adrenergic Sedation

Ying Ma,[1] Giulia Miracca,[1] Xiao Yu,[1] Edward C. Harding,[1] Andawei Miao,[1,2] Raquel Yustos,[1] Alexei L. Vyssotski,[3] Nicholas P. Franks,[1,2,*] and William Wisden[1,2,4,*]
[1]Department of Life Sciences, Imperial College, London SW7 2AZ, UK
[2]UK Dementia Research Institute, Imperial College, London SW7 2AZ, UK
[3]Institute of Neuroinformatics, University of Zurich and ETH Zurich, Zurich 8057, Switzerland
[4]Lead Contact
*Correspondence: n.franks@imperial.ac.uk (N.P.F.), w.wisden@imperial.ac.uk (W.W.)
https://doi.org/10.1016/j.cub.2019.07.087

SUMMARY

Our urge to sleep increases with time spent awake, until sleep becomes inescapable. The sleep following sleep deprivation is longer and deeper, with an increased power of delta (0.5–4 Hz) oscillations, a phenomenon termed sleep homeostasis [1–4]. Although widely expressed genes regulate sleep homeostasis [1, 4–10] and the process is tracked by somnogens and phosphorylation [1, 3, 7, 11–14], at the circuit level sleep homeostasis has remained mysterious. Previously, we found that sedation induced with α2-adrenergic agonists (e.g., dexmedetomidine) and sleep homeostasis both depend on the preoptic (PO) hypothalamus [15, 16]. Dexmedetomidine, increasingly used for long-term sedation in intensive care units [17], induces a non-rapid-eye-movement (NREM)-like sleep but with undesirable hypothermia [18, 19]. Within the PO, various neuronal subtypes (e.g., GABA/galanin and glutamate/NOS1) induce NREM sleep [20–22] and concomitant body cooling [21, 22]. This could be because NREM sleep's restorative effects depend on lower body temperature [23, 24]. Here, we show that mice with lesioned PO galanin neurons have reduced sleep homeostasis: in the recovery sleep following sleep deprivation there is a diminished increase in delta power, and the mice catch up little on lost sleep. Furthermore, dexmedetomidine cannot induce high-power delta oscillations or sustained hypothermia. Some hours after dexmedetomidine administration to wild-type mice there is a rebound in delta power when they enter normal NREM sleep, reminiscent of emergence from torpor. This delta rebound is reduced in mice lacking PO galanin neurons. Thus, sleep homeostasis and dexmedetomidine-induced sedation require PO galanin neurons and likely share common mechanisms.

RESULTS AND DISCUSSION

Ablating Galanin Neurons in the PO Hypothalamus

Ablating preoptic (PO) subnuclei produces insomnia and higher-amplitude diurnal oscillations in body temperature [25]. To selectively ablate PO^{Gal} neurons, we bilaterally injected an AAV mixture expressing Cre-activatable caspase-3 (AAV-*FLEX-CASP3*) and GFP (AAV-*FLEX-GFP*) transgenes into the lateral preoptic (LPO) area of *Gal-Cre* mice, generating *LPO-ΔGal* mice (Figures S1A–S1D). (Note: in the *Gal-Cre* mouse line [26], 95% of PO galanin-expressing neurons co-express Cre, and 95% of PO Cre-expressing cells co-express galanin [27].) As controls, *Gal-Cre* mice were injected only with AAV-*FLEX-GFP* to generate *LPO-Gal-GFP* mice (Figure S1A). In the *LPO-ΔGal* mouse group, immunohistochemistry with GFP antibodies showed that, after 5 weeks, the AAV-*FLEX-CASP3* injections eliminated ~98% of LPO^{Gal} cells, as compared with *LPO-Gal-GFP* littermate controls (Figures S1B–S1D). Numbers of parvalbumin-expressing cells (a neuronal population not expressing galanin [28]) in LPO were unaffected by caspase deletion of galanin neurons (Figure S1E), implying that the caspase selectively killed galanin neurons.

PO Galanin Neurons Are Needed for Consolidated NREM Sleep

To induce non-rapid-eye-movement (NREM) sleep, PO GABAergic neurons are believed to inhibit wake-promoting histamine neurons in the posterior hypothalamus [20, 29, 30] and express galanin [31]; indeed, galanin reduces the firing rate of histamine neurons [32]. There are, however, conflicting data on the consequences of activating PO galanin neurons. Optogenetic stimulation of PO galanin neuron soma at 10 Hz produced wakefulness [30]; however, lower stimulation frequencies (0.5–4 Hz) induced NREM sleep [21]. Chemogenetic activation of LPO galanin neurons also induced NREM sleep [21]—the authors of [21] found opto-stimulation frequencies above 8 Hz induced conduction block and inhibited PO galanin neurons, producing wake [21]. On the other hand, given that galanin neuronal subtypes exist [28], some could produce sleep, others wake.

To approach this issue from a complementary angle, we analyzed the 24-h sleep-wake cycle in *LPO-ΔGal* mice (Figures 1 and S2). Ablation of LPO galanin neurons modestly reduced

This is an open access article under the CC BY license (http://creativecommons.org/licenses/by/4.0/).

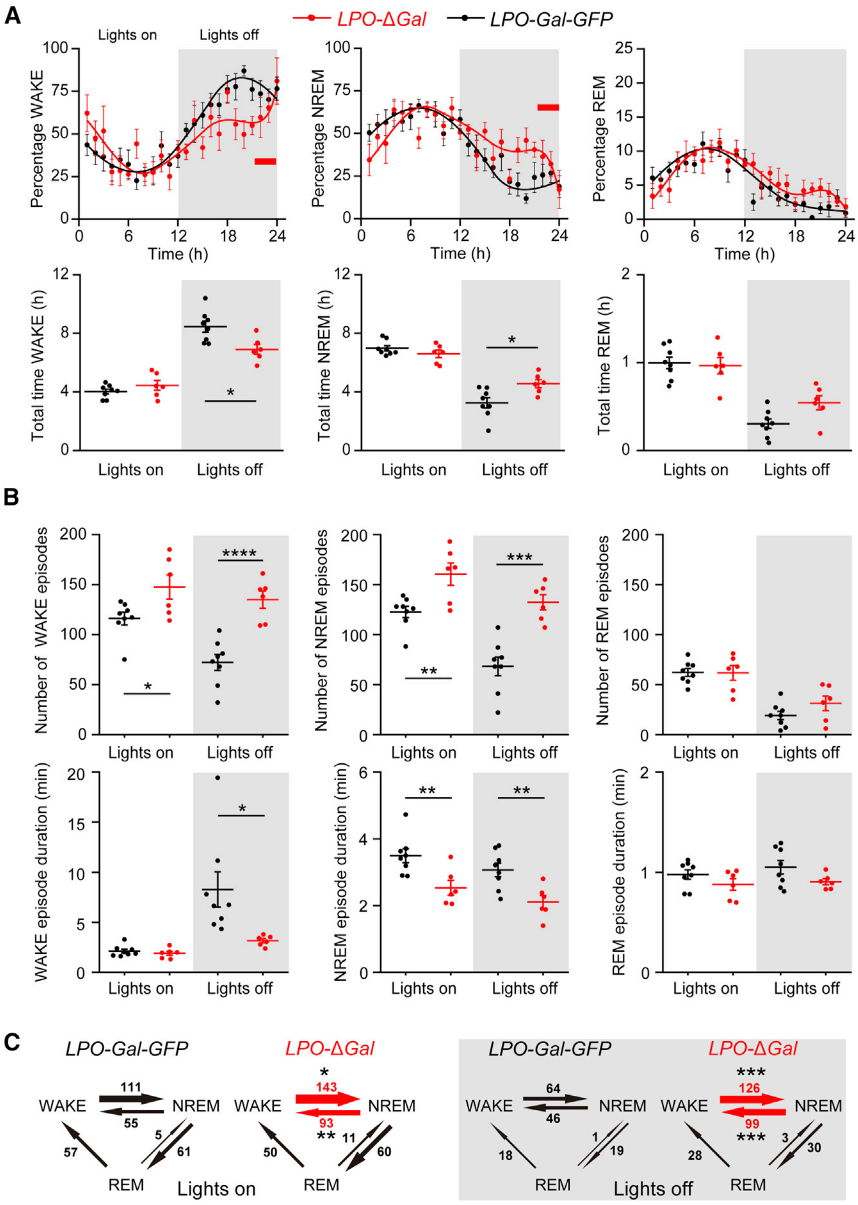

Figure 1. Ablation of Galanin Neurons in the LPO Caused Profound Fragmentation in Sleep Architecture

(A) Ablation of galanin neurons (see Figure S1) caused a modest reduction in total WAKE time (*$p < 0.05$, unpaired two-tailed t test) and an increase in total NREM time (*$p < 0.05$, unpaired two-tailed t test) during "lights off," but no change during "lights on." The amount of REM sleep was unaffected.

(B) Sleep architecture was highly fragmented by galanin neuron ablation. The number of WAKE and NREM episodes increased markedly, whereas their durations were shortened. These effects were most marked during lights off. The numbers of REM episodes and their durations were not affected. Example EEG and electromyogram (EMG) spectra are shown in Figure S2.

(C) The number of WAKE-to-NREM and NREM-to-WAKE transitions was significantly altered, but transitions between other vigilance states did not change (*LPO-Gal-GFP*, n = 8; *LPO-ΔGal*, n = 6). *$p < 0.05$, **$p < 0.01$, ***$p < 0.001$, ****$p < 0.0001$. All error bars represent the SEM. See also Figures S1–S3.

total wake time and increased total NREM time. These effects were specific for "lights off," the most active phase of the mice. There was no change in amounts of WAKE/NREM during "lights on" (Figure 1A). REM sleep was unaffected in either lights on or lights off. Furthermore, there were no differences in electroencephalogram (EEG) power between *LPO-Gal-GFP* and *LPO-ΔGal* mice in either the WAKE or NREM states (Figure S2B). Sleep architecture, however, was highly fragmented in *LPO-ΔGal* mice (Figure 1B). The number of WAKE and NREM episodes increased markedly, whereas their durations shortened. These effects were most marked during the lights off period. REM sleep episodes and their durations were not affected (Figure 1B). WAKE-to-NREM and NREM-to-WAKE transitions were significantly increased (Figure 1C), but transitions between other vigilance states did not change. Thus, even allowing for the high sleep-wake fragmentation that appears in *LPO-ΔGal* mice, LPO galanin neurons are dispensable for achieving NREM sleep. Nevertheless, acute chemogenetic activation of LPO^{Gal} neurons with clozapine-*N*-oxide (CNO) in *LPO-Gal-hM$_3$D$_q$* mice induced NREM sleep (Figures S3A–S3C), in agreement with earlier reports [21]. The delta power of this CNO-induced NREM sleep in *LPO-Gal-hM$_3$D$_q$* mice was higher than the power of NREM sleep after saline injection (Figure S3D). CNO had no effect on sleep in control mice (Figure S3E). Thus, galanin neurons can induce NREM sleep acutely (as first determined in [21]), but in the galanin-lesioned mice, it seems likely that other types of sleep-promoting neurons in the PO and elsewhere still induce sleep [22, 30]; however, galanin neurons are essential for consolidated sleep. Consistently, numbers of galanin neurons in the post-mortem human PO hypothalamus inversely correlate with the degree of sleep-wake fragmentation [33].

PO Galanin Neurons Chronically Lower Body Temperature

Certain PO neurons (e.g., GABA/galanin-, glutamate/NOS1-, PACAP/BDNF-, and TRPM2-expressing cells) respond to immediate external or internal thermal challenge by acutely initiating body cooling or heating [21, 22, 34–39], but it is unclear whether these cells are chronically regulating body temperature. Five weeks after ablation, *LPO-ΔGal* mice had a striking increase in their core body temperatures compared with *LPO-Gal-GFP* mice (Figures 2A and 2B). In a continuous 5-day recording of body temperature, the mice retained a diurnal variation of body

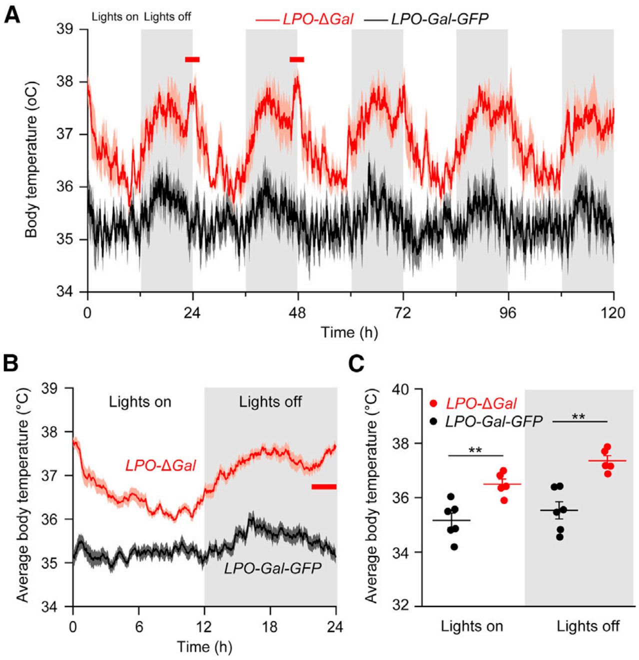

Figure 2. Chronic Ablation of LPO Galanin Neurons Markedly Elevated Core Body Temperature

(A) Ablation of LPO^{Gal} neurons caused increases in both the average core body temperature and its diurnal variation. The record shows typical recordings over 5 days for both *LPO-ΔGal* mice (red) and control *LPO-Gal-GFP* mice (black).

(B) Average core body temperature over 24 h (*LPO-ΔGal* mice and control *LPO-Gal-GFP* mice) also shows an abrupt and transient increase in body temperature around the transition from lights off to lights on in the *LPO-ΔGal* mice but not the control *LPO-Gal-GFP* mice.

(C) Average core body temperature increased in *LPO-ΔGal* mice in both lights on and lights off (**$p < 0.01$, unpaired two-tailed t test) (*LPO-Gal-GFP*, n = 6; *LPO-ΔGal*, n = 5). All error bars represent the SEM. See also Figure S2C, showing that locomotion was unaffected.

See also Figures S2 and S4.

temperature, with higher and lower temperatures during the lights off (active phase) and lights on (inactive phase) periods, respectively (Figures 2A and 2B). However, the average body temperature of *LPO-ΔGal* mice was raised to 37°C, compared with the average 35.5°C of *LPO-Gal-GFP* controls (Figure 2C). The raised body temperature of *LPO-ΔGal* mice was not explained by raised motor activity: *LPO-ΔGal* mice did not have changed locomotor activity in the open-field test (Figures S2C and S2D).

The diurnal range of body temperature change of *LPO-ΔGal* mice increased, reminiscent of the increase in amplitude of body temperature diurnal variation in PO-lesioned rats [25]. In the *LPO-Gal-GFP* control group, the average body temperature during the day and night was around 36°C and 35°C, respectively, whereas the *LPO-ΔGal* group had their average day and night body temperatures around 38°C and 36°C, respectively (Figure 2C). Thus, PO^{Gal} neurons act chronically to lower body temperature. This is supported by chemogenetic experiments, as first reported by others [21]. Indeed, we also found that chemogenetic activation of PO^{Gal} neurons with CNO in *LPO-Gal-hM$_3$D$_q$* mice induced hypothermia (Figures S3A, S4B, and S4C). PO galanin neurons are likely to initiate and maintain body cooling via the dorsomedial hypothalamus, rostral raphe pallidus, and rostroventral lateral medulla [38] pathways, which stop brown fat thermogenesis and induce blood vessel dilation.

A new feature emerged in the diurnal core body temperature variation in *LPO-ΔGal* mice: a pronounced positive spike appeared in their body temperature around the transition from lights off to lights on at zeitgeber time (ZT)24 (see red bars in Figures 2A and 2B), suggesting that LPO galanin neurons would normally lower body temperature at the point in the diurnal cycle where sleep pressure is highest at the start of the lights on period. Given that sleep induction occurs when the rate of core body temperature decline is maximal [40, 41], this could explain why *LPO-ΔGal* mice are actually more awake compared with *LPO-Gal-GFP* mice at the start of lights on (Figure 1A).

Although NREM sleep coincides with body cooling, a common perception is that during fever, when we are hot, we sleep more. The circuitry producing sleepiness in fever likely involves the PO [42]. Because *LPO-ΔGal* mice have higher temperatures, a hyperthermia-induced sleep might explain their total NREM sleep being increased from control mice, when in fact we might have expected total sleep time in *LPO-ΔGal* mice to decrease (as a result of losing sleep-promoting galanin neurons). A minimal conclusion, however, is that the NREM sleep occurring during hyperthermia does not require PO galanin neurons. But is hyperthermia, in any case, NREM sleep promoting? In one study, administration to mice of fever-promoting agents (e.g., TNFα and interleukin 1β) caused 12 h of hypothermia with increased amounts of NREM sleep, followed by 12 h of hyperthermia with normal amounts of NREM sleep (see Figure S1 in [43]). Furthermore, in rats, an increase of core body temperature from 37°C to 38°C correlated with decreased sleep time [44]. Finally, in our experiments, during the lights on phase, when sleep drive is highest, *LPO-ΔGal* mice have raised core body temperatures (Figure 2C) but have similar amounts of NREM sleep as control *LPO-Gal-GFP* mice (Figure 1A). Collectively, these examples show that hyperthermia per se does not necessarily increase NREM sleep.

PO Galanin Neurons Contribute to Sleep Homeostasis

The sleep following sleep deprivation is longer and deeper, with an increased power of delta (0.5–4 Hz) oscillations, a phenomenon termed sleep homeostasis [1–4]. At the circuit level, sleep homeostasis is partly regulated in the PO area [15, 45, 46], as well as locally in the neocortex [47–49]. To examine how LPO^{Gal} neurons regulate sleep homeostasis, a 5-h sleep deprivation was applied to both groups of mice. In control *LPO-Gal-GFP* mice, there was a reduction in wakefulness and an increase in total sleep (NREM + REM sleep) following 5 h of sleep deprivation (Figure 3A). The main effect, compared to the diurnal variation in wake and sleep times, occurred during the lights off period following sleep deprivation (which was carried out during the lights on period) (Figure 3A). In *LPO-ΔGal* mice, however, there was no change in WAKE or total sleep (NREM + REM) time following 5 h of sleep deprivation (Figure 3B). Most (~80%) of the sleep lost as a result of 5 h of sleep deprivation was recovered after 19 h in *LPO-Gal-GFP* mice, whereas only ~22% of the sleep loss was recovered in *LPO-ΔGal* mice (Figures 3C and 3D). Indeed, the sleep recovery rate after sleep deprivation

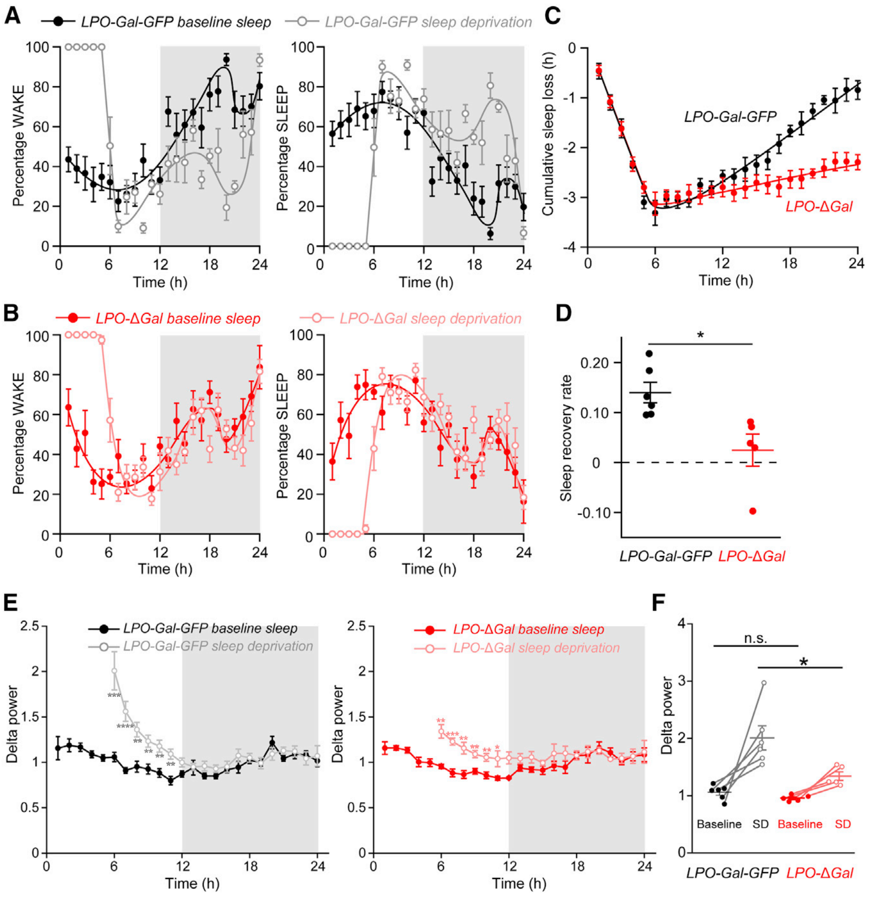

Figure 3. Homeostatic Sleep Rebound Following Sleep Deprivation Was Largely Abolished by the Ablation of LPO Galanin Neurons

(A) In control *LPO-Gal-GFP* mice, there was a strong reduction in WAKE and an increase in total sleep (NREM + REM) following 5 h of sleep deprivation. The main effect, compared to the diurnal variation in WAKE and sleep times, occurred during the lights off period following sleep deprivation (which was carried out during the lights on period) (n = 6).

(B) In *LPO-ΔGal* mice, however, there was no change in WAKE or total sleep (NREM + REM) time following 5 h of sleep deprivation (n = 5).

(C) Most (~80%) of the sleep lost as a result of 5 h of sleep deprivation was recovered after 19 h in *LPO-Gal-GFP* mice (black; n = 6), whereas only ~22% of the sleep loss was recovered in *LPO-ΔGal* mice (red; n = 5).

(D) The sleep recovery rate after sleep deprivation is significantly reduced in *LPO-ΔGal* mice (red; n = 5) compared with *LPO-Gal-GFP* (black; n = 6) (*$p < 0.05$, unpaired two-tailed t test).

(E) Time course of the delta power (0.5–4 Hz) after 5 h of sleep deprivation in *LPO-Gal-GFP* and *LPO-ΔGal* mice. In *LPO-Gal-GFP*, there was a large increase in delta power after sleep deprivation that decayed over 6 h back to baseline. By contrast, there was a lower increase in delta power after sleep deprivation in *LPO-ΔGal* mice (*$p < 0.05$, **$p < 0.01$, ***$p < 0.001$, ****$p < 0.0001$, repeated-measures one-way ANOVA with Holm-Bonferroni post-hoc test).

(F) Delta power (0.5–4 Hz) in the first hour of recovery sleep compared with baseline sleep in ZT6 for both *LPO-Gal-GFP* and *LPO-ΔGal* mice. *LPO-Gal-GFP* and *LPO-ΔGal* mice had similar delta power in their baseline sleep but, after 5 h of sleep deprivation, *LPO-Gal-GFP* mice had a larger increase in delta power compared with *LPO-ΔGal* mice (*$p < 0.05$, unpaired two-tailed t test) (*LPO-Gal-GFP*, n = 6; *LPO-ΔGal*, n = 5). n.s., not significant.

All error bars represent the SEM.

was significantly reduced in *LPO-ΔGal* mice compared with *LPO-Gal-GFP* (Figures 3C and 3D).

We also looked at delta power after sleep deprivation. The delta power of baseline NREM sleep varies characteristically over 24 h (see mouse data in [50]). This delta power baseline was similar between non-sleep-deprived *LPO-Gal-GFP* and *LPO-ΔGal* mice (Figure 3E). After 5-h sleep deprivation, during the first part of the sleep rebound of *LPO-Gal-GFP* mice, the power in the delta wave band was, as expected, increased compared to the delta power during sleep at the equivalent ZT (Figures 3E and 3F) (see the equivalent data in [50]). For the sleep-deprived *LPO-Gal-GFP* mice, the initial sharply increased delta power declined over the first 6 h post-sleep deprivation, until by the start of lights off it was approximately the same as baseline (Figure 3E). The increase in NREM delta power after sleep deprivation, although still significantly above the delta power baseline, was significantly reduced in *LPO-ΔGal* compared with *LPO-Gal-GFP* mice (Figures 3E and 3F). Our results suggest that sleep homeostasis can be regulated globally, via the hypothalamus. Another interpretation is that the sleep homeostasis of *LPO-ΔGal* mice is intact but the capacity of entering deep consolidated sleep with high delta power is deficient, for example as a result of elevated arousal-promoting drive; but because *LPO-ΔGal* mice are actually sleeping slightly more during their active lights off period (Figure 1A), this tends to suggest that their arousal-promoting drive is, if anything, diminished.

α2-Adrenergic Agonist-Induced Sedation and Hypothermia Require PO Galanin Neurons

Hypothermia is an unwanted side effect of dexmedetomidine [15, 19]. Indeed, in control *LPO-Gal-GFP* mice, after injection (intraperitoneal; i.p.) 50 μg/kg dexmedetomidine, there was a strong reduction in core body temperature from about 36°C to 25°C over the course of 2 h (post-dexmedetomidine injection) (Figures 4A and 4B). This hypothermia persisted beyond 4 h post-injection. In *LPO-ΔGal* mice, however, the initial reduction in body temperature after dexmedetomidine injection lasted only for the first hour and did not reach the same nadir as in *LPO-Gal-GFP* control mice, and the body temperature returned to normal levels over the next hour (Figures 4A and 4B). That the initial phase of body cooling triggered by dexmedetomidine still happens in the *LPO-ΔGal* mice could be because α2A receptors are found on smooth muscle of peripheral blood vessels and so dexmedetomidine will promote heating loss directly by vasodilation of tail veins.

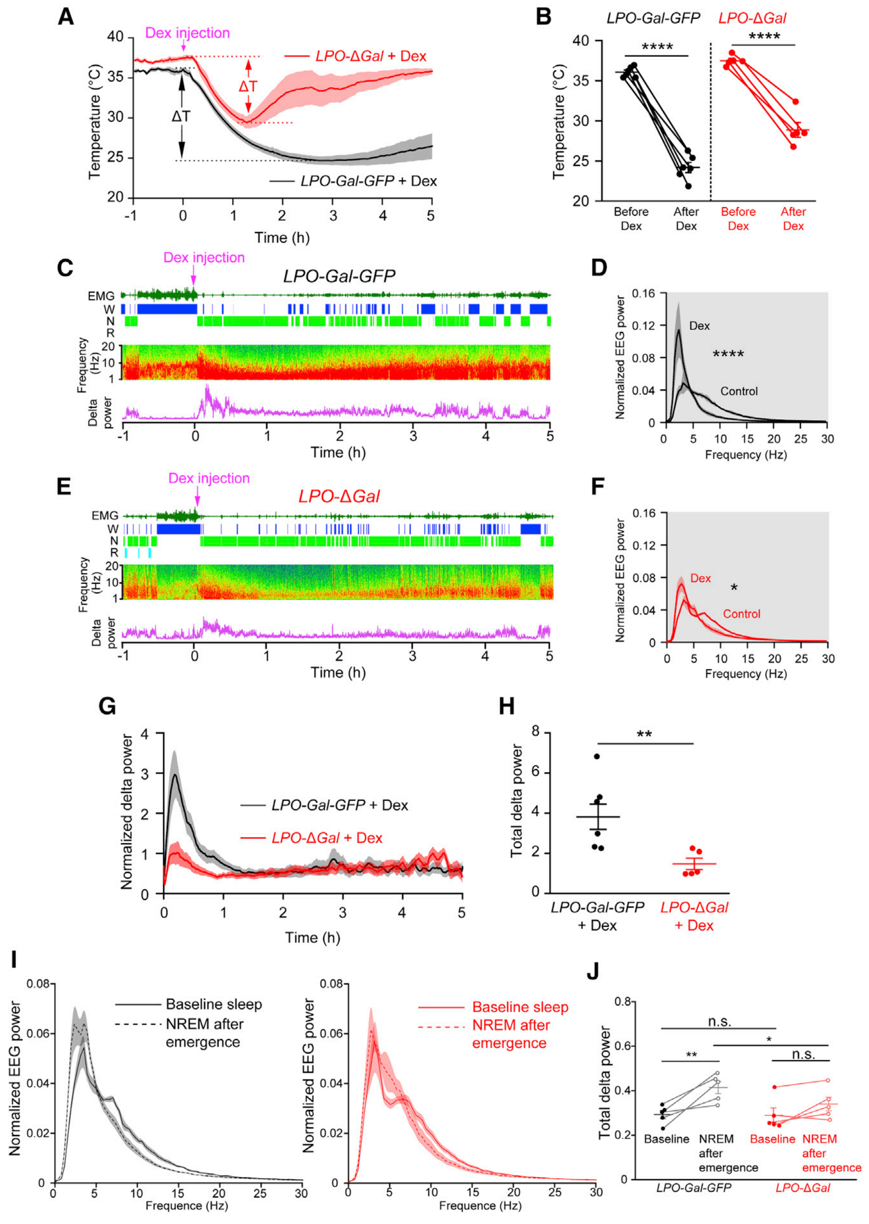

Figure 4. The Characteristic Sustained Hypothermia and Increased NREM-like Delta Power Induced by Dexmedetomidine Were Both Largely Abolished by the Ablation of LPO Galanin Neurons

(A) Dexmedetomidine was delivered at ZT19 (lights off), when mice were in their most active phase. In control *LPO-Gal-GFP* mice, there was a strong reduction in temperature from about 36°C to approximately 25°C over the course of 75 min (post-dexmedetomidine induction). This hypothermia persisted beyond 4 h post-injection. In *LPO-ΔGal* mice, however, the initial reduction in body temperature commenced after dexmedetomidine injection lasted only for the first hour and did not reach the same nadir as in *LPO-Gal-GFP* control mice, and the body temperature almost returned to starting levels (33°C ± 1.5°C) over the next hour.

(B) The body temperature before, and lowest temperature after, dexmedetomidine injection in *LPO-Gal-GFP* (****$p < 0.0001$, paired two-tailed t test; n = 6) and *LPO-ΔGal* mice (****$p < 0.0001$, paired two-tailed t test; n = 5).

(C) Dexmedetomidine injection of *LPO-Gal-GFP* mice: examples of EEG and EMG raw data and vigilance-state scoring.

(D) EEG power spectra of *LPO-Gal-GFP* mice, averaged over 30 min after dexmedetomidine injections. Dexmedetomidine induced a large increase in delta power relative to that of natural NREM sleep (control spectrum) (****$p < 0.0001$, paired two-tailed t test; n = 6).

(E) Dexmedetomidine injection into *LPO-ΔGal* mice: examples of EEG and EMG raw data and vigilance-state scoring.

(F) EEG power spectra of *LPO-ΔGal* mice, averaged over 30 min after dexmedetomidine injection. Dexmedetomidine induced a small increase in delta power relative to that of natural NREM sleep (control spectrum) (*$p < 0.05$, paired two-tailed t test; n = 5).

(G) Time courses of evoked NREM-like delta power following dexmedetomidine i.p. administration at ZT19 to *LPO-Gal-GFP* (black; n = 6) and *LPO-ΔGal* mice (red; n = 5).

(H) In *LPO-Gal-GFP* control mice (black; n = 6), within 20 min of injection, dexmedetomidine induced a large increase in delta power relative to that of NREM sleep, but this was substantially less in *LPO-ΔGal* mice (red; n = 5) (**$p < 0.01$, unpaired two-tailed t test).

(I) 3.5 h after *LPO-Gal-GFP* mice (black; n = 5) were given 50 μg/kg dexmedetomidine, the delta power band in NREM sleep was elevated compared with that of baseline NREM sleep at the corresponding circadian time. This effect was not present in *LPO-ΔGal* mice (red; n = 5).

(J) Quantification of delta rebound effect 3.5 h after dexmedetomidine in *LPO-Gal-GFP* mice and its absence in *LPO-DGal* mice (*$p < 0.05$, **$p < 0.01$; two-way ANOVA and Holm-Bonferroni post hoc test).

All error bars represent the SEM.

We investigated whether ablating LPO^{Gal} neurons compromised dexmedetomidine's ability to induce an NREM-like sleep state (Figures 4C–4F). After 1 h of EEG and temperature recordings, animals received 50 μg/kg dexmedetomidine (i.p.) in the lights off period, their active phase. In *LPO-Gal-GFP* control mice, within 20 min of injection, dexmedetomidine induced a large increase in delta power relative to that in NREM sleep occurring at the same ZT (Figures 4C and 4D), but this increase was substantially weaker in *LPO-ΔGal* mice (Figures 4E and 4F). (Note: REM sleep was nearly abolished after dexmedetomidine administration to both *LPO-Gal-GFP* and *LPO-ΔGal* groups.) Looking at the time course of the evoked delta power following dexmedetomidine injection (Figures 4G and 4H), in *LPO-Gal-GFP* mice the delta power peaked at 11 min post-injection and then declined over the following hours even though the mice were still in an NREM-like sleep. (Because of the evolving hypothermia in the dexmedetomidine-injected *LPO-Gal-GFP* mice [Figure 4A], the power of the EEG spectrum declines with time

as we and others documented previously; however, the vigilance state can still be scored as NREM sleep-like [22, 51].) By contrast, the delta power remained at normal NREM sleep levels in dexmedetomidine-injected *LPO-ΔGal* mice, even at the start of the experiment (Figures 4G and 4H). (If wild-type animals that are given dexmedetomidine are kept warm, the high NREM-like delta power that is triggered by the drug does not diminish [unpublished data].)

Because daily torpor in some species leads to a rebound of delta power during subsequent NREM sleep [52, 53], we asked by analogy whether dexmedetomidine also caused a delta power rebound. Indeed, looking at NREM sleep 3.5 h after dexmedetomidine injection, there was a rebound in delta power when baseline NREM sleep resumed after dexmedetomidine injection. The rebound was absent in *LPO-ΔGal* mice (Figures 4I and 4J), highlighting a further link between the mechanism of dexmedetomidine-induced sedation and the sleep homeostasis machinery.

The metabotropic α2A-adrenergic receptor mediates both the NREM sleep-like state and the hypothermic effects of dexmedetomidine [54, 55]. Although it is commonly thought that dexmedetomidine induces sedation by inhibiting noradrenaline release from neurons in the locus ceruleus [55–57], there is evidence that this is not the case [15, 18, 58]: VLPO lesions in rats blunt the ability of dexmedetomidine to induce sedation [16]; dexmedetomidine induces cFOS expression in the PO [15, 16], and can induce sedation even when noradrenaline release from the locus ceruleus is genetically removed [58]; and reactivating ensembles of PO neuron activity tagged during dexmedetomidine-induced is sufficient to induce NREM sleep and hypothermia [15]. Dexmedetomidine is expected to directly excite PO neurons by influencing hyperpolarization-activated cyclic nucleotide-gated cation channels [59].

Subtypes of PO Galanin Neurons

In the simplest case, one PO galanin neuron type, sensitive to the factors driving sleep homeostasis and having α2A receptors, induces and maintains NREM sleep and body cooling. This seems unlikely. In fact, single-cell profiling and multiplex *in situ* labeling found multiple subtypes of galanin-expressing neuron, dispersed and intermingled, in the mouse PO region [28]. Most of these galanin neurons are GABAergic, but several are glutamatergic, and one *vgat*/galanin subtype expresses tyrosine hydroxylase and the vesicular monoamine transporter [28]. To untangle the circuit logic, intersectional genetics combined with c-FOS-based activity tagging could be deployed [22].

Conclusions

PO galanin neurons are required for consolidated NREM sleep, and mediate both rebound NREM sleep linked to body cooling and adrenergic agonist-induced sedation with sustained hypothermia. PO galanin neuronal activation causes higher delta power (i.e., a more synchronized state of neocortical activity), whether through sleep homeostasis following sleep deprivation or chemogenetic and dexmedetomidine activation. We and others have suggested that strengthening this sleep and cooling process, triggered through, e.g., PO glutamate/NOS1 neurons and galanin neurons, could cause torpor [21, 22]. It seems likely that dexmedetomidine, through galanin neurons, overactivates the natural sleep homeostasis pathway, pushing the animal into deep hypothermia, from which there is a subsequent delta NREM rebound. This suggests that the dexmedetomidine-induced sedation is not restorative like normal sleep, possibly because the mice become too cold. Identifying the cellular substrates of dexmedetomidine's sedative actions could help discover drugs causing strong sedation without excessive cooling.

STAR★METHODS

Detailed methods are provided in the online version of this paper and include the following:

- KEY RESOURCES TABLE
- LEAD CONTACT AND MATERIALS AVAILABILITY
- EXPERIMENTAL MODEL AND SUBJECT DETAILS
  - Mice
- METHOD DETAILS
  - AAV transgene plasmids
  - Generation of recombinant AAV particles
  - Surgeries and stereotaxic injection of AAV
  - EEG and EMG recordings and vigilance states scoring
  - Core body temperature recording
  - Sleep deprivation and recovery sleep
  - Chemogenetics and behavioral assessment
  - Dexmedetomidine experiments
  - Immunohistochemistry
- QUANTIFICATION AND STATISTICAL ANALYSIS
- DATA AND CODE AVAILABILITY

SUPPLEMENTAL INFORMATION

Supplemental Information can be found online at https://doi.org/10.1016/j.cub.2019.07.087.

ACKNOWLEDGMENTS

Our work was supported by the Wellcome Trust (107839/Z/15/Z, N.P.F.; 107841/Z/15/Z, W.W.), the UK Dementia Research Institute (W.W. and N.P.F.), an Imperial College London and China Scholarship Council scholarship (Y.M.), an Imperial College Schrödinger scholarship (G.M.), and a studentship from the UK Medical Research Council (A.M.). The Facility for Imaging by Light Microscopy (FILM) at Imperial College London is in part supported by funding from the Wellcome Trust (grant 104931/Z/14/Z) and BBSRC (grant BB/L015129/1).

AUTHOR CONTRIBUTIONS

Conceptualization, N.P.F. and W.W.; Investigation, Y.M., G.M., X.Y., E.C.H., A.M., and R.Y.; Writing – Original Draft, Y.M., N.P.F., and W.W.; Writing – Review & Editing, Y.M., N.P.F., and W.W.; Funding Acquisition, N.P.F. and W.W.; Resources, A.L.V.; Supervision, N.P.F. and W.W.

DECLARATION OF INTERESTS

The authors declare no competing interests.

Received: March 24, 2019
Revised: May 24, 2019
Accepted: July 30, 2019
Published: September 19, 2019

REFERENCES

1. Greene, R.W., Bjorness, T.E., and Suzuki, A. (2017). The adenosine-mediated, neuronal-glial, homeostatic sleep response. Curr. Opin. Neurobiol. *44*, 236–242.
2. Borbély, A.A., Daan, S., Wirz-Justice, A., and Deboer, T. (2016). The two-process model of sleep regulation: a reappraisal. J. Sleep Res. *25*, 131–143.
3. Porkka-Heiskanen, T. (2013). Sleep homeostasis. Curr. Opin. Neurobiol. *23*, 799–805.
4. Franken, P., Chollet, D., and Tafti, M. (2001). The homeostatic regulation of sleep need is under genetic control. J. Neurosci. *21*, 2610–2621.
5. Funato, H., Miyoshi, C., Fujiyama, T., Kanda, T., Sato, M., Wang, Z., Ma, J., Nakane, S., Tomita, J., Ikkyu, A., et al. (2016). Forward-genetics analysis of sleep in randomly mutagenized mice. Nature *539*, 378–383.
6. Holst, S.C., Sousek, A., Hefti, K., Saberi-Moghadam, S., Buck, A., Ametamey, S.M., Scheidegger, M., Franken, P., Henning, A., Seifritz, E., et al. (2017). Cerebral mGluR5 availability contributes to elevated sleep need and behavioral adjustment after sleep deprivation. eLife *6*, e28751.
7. Bjorness, T.E., Kelly, C.L., Gao, T., Poffenberger, V., and Greene, R.W. (2009). Control and function of the homeostatic sleep response by adenosine A1 receptors. J. Neurosci. *29*, 1267–1276.
8. Franken, P. (2013). A role for clock genes in sleep homeostasis. Curr. Opin. Neurobiol. *23*, 864–872.
9. Mang, G.M., La Spada, F., Emmenegger, Y., Chappuis, S., Ripperger, J.A., Albrecht, U., and Franken, P. (2016). Altered sleep homeostasis in Rev-erbα knockout mice. Sleep (Basel) *39*, 589–601.
10. Hasan, S., van der Veen, D.R., Winsky-Sommerer, R., Hogben, A., Laing, E.E., Koentgen, F., Dijk, D.J., and Archer, S.N. (2014). A human sleep homeostasis phenotype in mice expressing a primate-specific PER3 variable-number tandem-repeat coding-region polymorphism. FASEB J. *28*, 2441–2454.
11. Halassa, M.M., Florian, C., Fellin, T., Munoz, J.R., Lee, S.Y., Abel, T., Haydon, P.G., and Frank, M.G. (2009). Astrocytic modulation of sleep homeostasis and cognitive consequences of sleep loss. Neuron *61*, 213–219.
12. Leenaars, C.H.C., Savelyev, S.A., Van der Mierden, S., Joosten, R.N.J.M.A., Dematteis, M., Porkka-Heiskanen, T., and Feenstra, M.G.P. (2018). Intracerebral adenosine during sleep deprivation: a meta-analysis and new experimental data. J. Circadian Rhythms *16*, 11.
13. Porkka-Heiskanen, T., Strecker, R.E., Thakkar, M., Bjorkum, A.A., Greene, R.W., and McCarley, R.W. (1997). Adenosine: a mediator of the sleep-inducing effects of prolonged wakefulness. Science *276*, 1265–1268.
14. Wang, Z., Ma, J., Miyoshi, C., Li, Y., Sato, M., Ogawa, Y., Lou, T., Ma, C., Gao, X., Lee, C., et al. (2018). Quantitative phosphoproteomic analysis of the molecular substrates of sleep need. Nature *558*, 435–439.
15. Zhang, Z., Ferretti, V., Güntan, İ., Moro, A., Steinberg, E.A., Ye, Z., Zecharia, A.Y., Yu, X., Vyssotski, A.L., Brickley, S.G., et al. (2015). Neuronal ensembles sufficient for recovery sleep and the sedative actions of α2 adrenergic agonists. Nat. Neurosci. *18*, 553–561.
16. Nelson, L.E., Lu, J., Guo, T., Saper, C.B., Franks, N.P., and Maze, M. (2003). The alpha2-adrenoceptor agonist dexmedetomidine converges on an endogenous sleep-promoting pathway to exert its sedative effects. Anesthesiology *98*, 428–436.
17. Adams, R., Brown, G.T., Davidson, M., Fisher, E., Mathisen, J., Thomson, G., and Webster, N.R. (2013). Efficacy of dexmedetomidine compared with midazolam for sedation in adult intensive care patients: a systematic review. Br. J. Anaesth. *111*, 703–710.
18. Yu, X., Franks, N.P., and Wisden, W. (2018). Sleep and sedative states induced by targeting the histamine and noradrenergic systems. Front. Neural Circuits *12*, 4.
19. Seidel, W.F., Maze, M., Dement, W.C., and Edgar, D.M. (1995). Alpha-2 adrenergic modulation of sleep: time-of-day-dependent pharmacodynamic profiles of dexmedetomidine and clonidine in the rat. J. Pharmacol. Exp. Ther. *275*, 263–273.
20. Sherin, J.E., Shiromani, P.J., McCarley, R.W., and Saper, C.B. (1996). Activation of ventrolateral preoptic neurons during sleep. Science *271*, 216–219.
21. Kroeger, D., Absi, G., Gagliardi, C., Bandaru, S.S., Madara, J.C., Ferrari, L.L., Arrigoni, E., Münzberg, H., Scammell, T.E., Saper, C.B., and Vetrivelan, R. (2018). Galanin neurons in the ventrolateral preoptic area promote sleep and heat loss in mice. Nat. Commun. *9*, 4129.
22. Harding, E.C., Yu, X., Miao, A., Andrews, N., Ma, Y., Ye, Z., Lignos, L., Miracca, G., Ba, W., Yustos, R., et al. (2018). A neuronal hub binding sleep initiation and body cooling in response to a warm external stimulus. Curr. Biol. *28*, 2263–2273.e4.
23. McGinty, D., and Szymusiak, R. (1990). Keeping cool: a hypothesis about the mechanisms and functions of slow-wave sleep. Trends Neurosci. *13*, 480–487.
24. Hoekstra, M.M., Emmenegger, Y., Hubbard, J., and Franken, P. (2019). Cold-inducible RNA-binding protein (CIRBP) adjusts clock-gene expression and REM-sleep recovery following sleep deprivation. eLife *8*, e43400.
25. Lu, J., Greco, M.A., Shiromani, P., and Saper, C.B. (2000). Effect of lesions of the ventrolateral preoptic nucleus on NREM and REM sleep. J. Neurosci. *20*, 3830–3842.
26. Schmidt, E.F., Kus, L., Gong, S., and Heintz, N. (2013). BAC transgenic mice and the GENSAT database of engineered mouse strains. Cold Spring Harb. Protoc. *2013*, pdb.top073692.
27. Wu, Z., Autry, A.E., Bergan, J.F., Watabe-Uchida, M., and Dulac, C.G. (2014). Galanin neurons in the medial preoptic area govern parental behaviour. Nature *509*, 325–330.
28. Moffitt, J.R., Bambah-Mukku, D., Eichhorn, S.W., Vaughn, E., Shekhar, K., Perez, J.D., Rubinstein, N.D., Hao, J., Regev, A., Dulac, C., and Zhuang, X. (2018). Molecular, spatial, and functional single-cell profiling of the hypothalamic preoptic region. Science *362*, eaau5324.
29. Saper, C.B., Scammell, T.E., and Lu, J. (2005). Hypothalamic regulation of sleep and circadian rhythms. Nature *437*, 1257–1263.
30. Chung, S., Weber, F., Zhong, P., Tan, C.L., Nguyen, T.N., Beier, K.T., Hörmann, N., Chang, W.C., Zhang, Z., Do, J.P., et al. (2017). Identification of preoptic sleep neurons using retrograde labelling and gene profiling. Nature *545*, 477–481.
31. Sherin, J.E., Elmquist, J.K., Torrealba, F., and Saper, C.B. (1998). Innervation of histaminergic tuberomammillary neurons by GABAergic and galaninergic neurons in the ventrolateral preoptic nucleus of the rat. J. Neurosci. *18*, 4705–4721.
32. Schönrock, B., Büsselberg, D., and Haas, H.L. (1991). Properties of tuberomammillary histamine neurones and their response to galanin. Agents Actions *33*, 135–137.
33. Lim, A.S., Ellison, B.A., Wang, J.L., Yu, L., Schneider, J.A., Buchman, A.S., Bennett, D.A., and Saper, C.B. (2014). Sleep is related to neuron numbers in the ventrolateral preoptic/intermediate nucleus in older adults with and without Alzheimer's disease. Brain *137*, 2847–2861.
34. Tan, C.L., and Knight, Z.A. (2018). Regulation of body temperature by the nervous system. Neuron *98*, 31–48.
35. Siemens, J., and Kamm, G.B. (2018). Cellular populations and thermosensing mechanisms of the hypothalamic thermoregulatory center. Pflugers Arch. *470*, 809–822.
36. Tan, C.L., Cooke, E.K., Leib, D.E., Lin, Y.C., Daly, G.E., Zimmerman, C.A., and Knight, Z.A. (2016). Warm-sensitive neurons that control body temperature. Cell *167*, 47–59.e15.
37. Song, K., Wang, H., Kamm, G.B., Pohle, J., Reis, F.C., Heppenstall, P., Wende, H., and Siemens, J. (2016). The TRPM2 channel is a hypothalamic heat sensor that limits fever and can drive hypothermia. Science *353*, 1393–1398.
38. Zhao, Z.D., Yang, W.Z., Gao, C., Fu, X., Zhang, W., Zhou, Q., Chen, W., Ni, X., Lin, J.K., Yang, J., et al. (2017). A hypothalamic circuit that controls body temperature. Proc. Natl. Acad. Sci. USA *114*, 2042–2047.

39. Nakamura, K., and Morrison, S.F. (2008). A thermosensory pathway that controls body temperature. Nat. Neurosci. *11*, 62–71.
40. Campbell, S.S., and Broughton, R.J. (1994). Rapid decline in body temperature before sleep: fluffing the physiological pillow? Chronobiol. Int. *11*, 126–131.
41. Harding, E.C., Franks, N.P., and Wisden, W. (2019). The temperature dependence of sleep. Front. Neurosci. *13*, 336.
42. Saper, C.B., Romanovsky, A.A., and Scammell, T.E. (2012). Neural circuitry engaged by prostaglandins during the sickness syndrome. Nat. Neurosci. *15*, 1088–1095.
43. Szentirmai, É., and Kapás, L. (2018). Brown adipose tissue plays a central role in systemic inflammation-induced sleep responses. PLoS ONE *13*, e0197409.
44. Kumar, D., Mallick, H.N., and Kumar, V.M. (2009). Ambient temperature that induces maximum sleep in rats. Physiol. Behav. *98*, 186–191.
45. Gong, H., McGinty, D., Guzman-Marin, R., Chew, K.T., Stewart, D., and Szymusiak, R. (2004). Activation of c-fos in GABAergic neurones in the preoptic area during sleep and in response to sleep deprivation. J. Physiol. *556*, 935–946.
46. Gvilia, I., Xu, F., McGinty, D., and Szymusiak, R. (2006). Homeostatic regulation of sleep: a role for preoptic area neurons. J. Neurosci. *26*, 9426–9433.
47. Huber, R., Ghilardi, M.F., Massimini, M., and Tononi, G. (2004). Local sleep and learning. Nature *430*, 78–81.
48. Siclari, F., and Tononi, G. (2017). Local aspects of sleep and wakefulness. Curr. Opin. Neurobiol. *44*, 222–227.
49. Guillaumin, M.C.C., McKillop, L.E., Cui, N., Fisher, S.P., Foster, R.G., de Vos, M., Peirson, S.N., Achermann, P., and Vyazovskiy, V.V. (2018). Cortical region–specific sleep homeostasis in mice: effects of time of day and waking experience. Sleep *41*, zsy079.
50. Huber, R., Deboer, T., and Tobler, I. (2000). Effects of sleep deprivation on sleep and sleep EEG in three mouse strains: empirical data and simulations. Brain Res. *857*, 8–19.
51. Deboer, T., and Tobler, I. (1995). Temperature dependence of EEG frequencies during natural hypothermia. Brain Res. *670*, 153–156.
52. Daan, S., Barnes, B.M., and Strijkstra, A.M. (1991). Warming up for sleep? Ground squirrels sleep during arousals from hibernation. Neurosci. Lett. *128*, 265–268.
53. Trachsel, L., Edgar, D.M., and Heller, H.C. (1991). Are ground squirrels sleep deprived during hibernation? Am. J. Physiol. *260*, R1123–R1129.
54. Hunter, J.C., Fontana, D.J., Hedley, L.R., Jasper, J.R., Lewis, R., Link, R.E., Secchi, R., Sutton, J., and Eglen, R.M. (1997). Assessment of the role of alpha2-adrenoceptor subtypes in the antinociceptive, sedative and hypothermic action of dexmedetomidine in transgenic mice. Br. J. Pharmacol. *122*, 1339–1344.
55. Lakhlani, P.P., MacMillan, L.B., Guo, T.Z., McCool, B.A., Lovinger, D.M., Maze, M., and Limbird, L.E. (1997). Substitution of a mutant alpha2a-adrenergic receptor via ‘‘hit and run’’ gene targeting reveals the role of this subtype in sedative, analgesic, and anesthetic-sparing responses in vivo. Proc. Natl. Acad. Sci. USA *94*, 9950–9955.
56. Correa-Sales, C., Rabin, B.C., and Maze, M. (1992). A hypnotic response to dexmedetomidine, an alpha 2 agonist, is mediated in the locus coeruleus in rats. Anesthesiology *76*, 948–952.
57. Sanders, R.D., and Maze, M. (2012). Noradrenergic trespass in anesthetic and sedative states. Anesthesiology *117*, 945–947.
58. Hu, F.Y., Hanna, G.M., Han, W., Mardini, F., Thomas, S.A., Wyner, A.J., and Kelz, M.B. (2012). Hypnotic hypersensitivity to volatile anesthetics and dexmedetomidine in dopamine β-hydroxylase knockout mice. Anesthesiology *117*, 1006–1017.
59. Harris, N.A., Isaac, A.T., Günther, A., Merkel, K., Melchior, J., Xu, M., Eguakun, E., Perez, R., Nabit, B.P., Flavin, S., et al. (2018). Dorsal BNST α_{2A}-adrenergic receptors produce HCN-dependent excitatory actions that initiate anxiogenic behaviors. J. Neurosci. *38*, 8922–8942.
60. Yang, C.F., Chiang, M.C., Gray, D.C., Prabhakaran, M., Alvarado, M., Juntti, S.A., Unger, E.K., Wells, J.A., and Shah, N.M. (2013). Sexually dimorphic neurons in the ventromedial hypothalamus govern mating in both sexes and aggression in males. Cell *153*, 896–909.
61. Krashes, M.J., Koda, S., Ye, C., Rogan, S.C., Adams, A.C., Cusher, D.S., Maratos-Flier, E., Roth, B.L., and Lowell, B.B. (2011). Rapid, reversible activation of AgRP neurons drives feeding behavior in mice. J. Clin. Invest. *121*, 1424–1428.
62. Klugmann, M., Symes, C.W., Leichtlein, C.B., Klaussner, B.K., Dunning, J., Fong, D., Young, D., and During, M.J. (2005). AAV-mediated hippocampal expression of short and long Homer 1 proteins differentially affect cognition and seizure activity in adult rats. Mol. Cell. Neurosci. *28*, 347–360.
63. Anisimov, V.N., Herbst, J.A., Abramchuk, A.N., Latanov, A.V., Hahnloser, R.H., and Vyssotski, A.L. (2014). Reconstruction of vocal interactions in a group of small songbirds. Nat. Methods *11*, 1135–1137.

STAR★METHODS

KEY RESOURCES TABLE

REAGENT or RESOURCE	SOURCE	IDENTIFIER
Antibodies		
Anti-EGFP rabbit polyclonal antibody	Thermo Fisher Scientific	A6455
Anti-mCherry mouse monoclonal antibody	Clontech	632543; RRID: AB_2307319
Rabbit polyclonal anti-parvalbumin	Abcam	ab11427
Alexa Flour 488 goat anti-rabbit IgG	Molecular Probes	A11034; RRID: AB_2576217
Alexa Flour 594 goat anti-mouse IgG	Molecular Probes	A11005; RRID: AB_141372
Chemicals, Peptides, and Recombinant Proteins		
Isoflurane	Zoetis	50019100
Clozapine *N*-Oxide	Tocris	4936
Dexmedetomidine	Tocris	2749
Experimental Models: Cell Lines		
HEK293 cells	Sigma-Aldrich	85120602/CVCL_0045
Experimental Models: Organisms/Strains		
Mouse: Tg(Gal-cre)KI87Gsat/Mmucd	Mutant Mouse Regional Resource Center	stock No. 031060-UCD
Oligonucleotides		
Galanin primers	Mutant Mouse Regional Resource Center	Primers as recommended
Recombinant DNA		
Adenovirus helper plasmid *pFΔ6*	Donated by M Klugmann	N/A
AAV helper plasmid *pH21* (AAV1)	Donated by M Klugmann	N/A
AAV helper plasmid pRVI (AAV2)	Donated by M Klugmann	N/A
pAAV-EF1α-flex-taCasp3-TEVp	Donated by Nirao Shah	Addgene #45580
pAAV-CAG-flex-GFP	Donated by Edward Boyden	Addgene #28304
pAAV-hSyn-flex-hM3Dq-mCherry	Donated by Bryan Roth	Addgene #44361
Software and Algorithms		
Spike2 v7.18	Cambridge Electronic Design	http://ced.co.uk/products/spkovin
MATLAB (Version R2016b)	MathWorks	https://uk.mathworks.com/
Downloader (Version 1.27)	Evolocus	http://www.evolocus.com
ImageJ	Fiji	https://fiji.sc/
Activity Monitor Version 5 for mice	Medical Associates	http://www.med-associates.com/product-category/activity-software/
Other		
1-ml HiTrap Heparin column	Sigma-Aldrich	5-4836
Angle Two stereotaxic frame	Leica Microsystems	N/A
Hamilton microliter 10-μl syringes	Hamilton	701
Neurologger 2A	Alexei L. Vyssotski	N/A
Temperature logger	STAR-ODDI	DST nano-T

LEAD CONTACT AND MATERIALS AVAILABILITY

Further information and requests for resources and reagents should be directed to, and will be fulfilled by, the Lead Contact, William Wisden (w.wisden@imperial.ac.uk). Please note that this study did not generate new unique reagents.

EXPERIMENTAL MODEL AND SUBJECT DETAILS

Mice

Animal care and experiments were performed under the UK Home Office Animal Procedures Act (1986) and were approved by the Imperial College Ethical Review Committee. *Gal-Cre* mice (Tg(Gal-cre)KI87Gsat/Mmucd) were generated by GENSAT and deposited

at the Mutant Mouse Regional Resource Center, stock No. 031060-UCD, The Gene Expression Nervous System Atlas (GENSAT) Project (NINDS Contracts N01NS02331 & HHSN271200723701C to The Rockefeller University, New York) [26]. In this mouse line, Cre recombinase expression is driven from a bacterial artificial chromosome transgene containing the endogenous mouse galanin gene. All mice used in the experiment were equally mixed genders and had the first surgery at the age of 10-12 weeks. Mice were housed individually. *Ad libitum* food and water were available for all mice and a reversed 12 h:12 h light/dark cycle (''lights on'' hours: 17:00-05:00) with constant temperature and humidity.

METHOD DETAILS

AAV transgene plasmids

AAV transgenes had a flexed reading frame in an inverted orientation, and therefore could only be activated by Cre recombinase. The *pAAV-EF1α-flex-taCasp3-TEVp* transgene plasmid was Addgene plasmid #45580 (a gift from Nirao Shah) [60]. The *pAAV-CAG-flex-GFP* transgene construct was Addgene plasmid #28304 (a gift from Edward Boyden). The *pAAV-hSyn-flex-hM*$_3$*D*$_q$*-mCherry* transgene construct was Addgene plasmid #44361 (a gift from Bryan Roth) [61].

Generation of recombinant AAV particles

All AAV transgenes were packaged in our laboratory into AAV capsids with a mixed serotype 1 & 2 (1:1 ratio of AAV1 and AAV2 capsid proteins) as described previously [62].

Surgeries and stereotaxic injection of AAV

For surgery, mice were anesthetized with an initiation concentration of 2.5% isoflurane in O_2 (vol/vol) by inhalation and mounted into a stereotaxic frame (Angle Two, Leica Microsystems, Milton Keynes, Buckinghamshire, UK). Mice were maintained anesthetized on 2% isoflurane during surgery. A heat pad was used during the whole surgery to prevent heat loss. For ablating galanin neurons, the two AAV viruses, AAV-*EF1α-flex-taCasp3-TEVp* and AAV-*CAG-flex-GFP* were mixed in a 1:1 ratio prior to injection while a single virus type was injected for the rest of experiments unless otherwise stated. For the parvalbumin quantification experiment in Figure S1E, AAV-*flex-mCherry* was injected instead of AAV-*CAG-flex-GFP*. AAV viruses were delivered using a 10 μL syringe (Hamilton microliter, #701) with a 33-gauge stainless steel needle (point style 3, length 1.5 cm, Hamilton). The injection coordinates (bilateral) for the LPO relative to Bregma were: AP +0.02 mm; ML ± 0.75 mm; DV was consecutive starting −5.8 (1/2 volume), −5.6 (1/2 volume). A total volume of 0.2-0.5 μL of virus was injected into each hemisphere depending on the viral titration. Mice were allowed three weeks for recovery in their home cage before fitting with Neurologger 2A devices (see section below) and performing behavioral experiments. For experiments where temperature recordings were necessary, temperature loggers were usually inserted (abdominally) two to three weeks after mice had had their viral injection surgeries.

EEG and EMG recordings and vigilance states scoring

Non-tethered EEG and EMG recordings were captured using Neurologger 2A devices [63]. Screw electrodes were chronically inserted into the skull of mice to measure cortical EEG using the following coordinates: −1.5 mm Bregma, + 1.5 mm midline - first recording electrode; + 1.5 mm Bregma, −1.5 mm midline – second recording electrode; −1 mm Lambda, 0 mm midline – reference electrode. EMG signals were recorded by a pair of stainless steel electrodes implanted in the dorsal neck muscle. Four data channels (2 of EEG and 2 of EMG) were recorded with four times oversampling at a sampling rate of 200 Hz. The dataset was downloaded and waveforms visualized using Spike2 software (Cambridge Electronic Design, Cambridge, UK) or MATLAB (MathWorks, Cambridge, UK). The EEG signals were high-pass filtered (0.5 Hz, −3dB, an FFT size of 512 was the designated time window) using a digital filter and the EMG was band-pass filtered between 5-45 Hz (−3dB). Power in the delta (0.5-4 Hz), theta (6-10 Hz) bands and theta to delta band ratio were calculated, along with the root mean square (RMS) value of the EMG signal (averaged over a bin size of 5 s). All of these data were used to define the vigilance states of WAKE, NREM and REM by an automatic script. Each vigilance state was screened and confirmed manually afterward. The peak frequency during NREM epochs were analyzed using Fourier transform power spectra to average power spectra over blocks of time.

Core body temperature recording

Core body temperature was recorded using temperature loggers (DST nano, Star-Oddi, HerfØlge, Denmark) implanted abdominally. A pre-defined program was set to sample the temperature data every two minutes for baseline core body temperature and drug/vehicle administration. At the end of the experiments, the loggers were retrieved and the data were downloaded and analyzed.

Sleep deprivation and recovery sleep

The sleep deprivation protocol was similar to the one we used before [15], and started at ZT zero (17:00), the start of the ''lights on'' period when the sleep drive of the mice is at its maximum. Both experimental and control groups were removed from their home cages and sleep deprived for 5 hours by introducing novel objects or gently tapping on the cages. Handling was kept to a minimum. After sleep deprivation, mice were immediately placed back in their home cages. EEG and EMG signals were recorded during the sleep deprivation and recovery periods.

Chemogenetics and behavioral assessment

For chemogenetic activation, clozapine-N-oxide (CNO) (C0832, Sigma-Aldrich) was used. 1 mg/kg of CNO dissolved in saline or saline in the same volume was administrated by intraperitoneal (*i.p.*) injection and the EEG/EMG data and core body temperature were recorded. Mice were split into random groups that either received CNO or saline injection for an unambiguous comparison. Drugs were administrated at ZT18 (11:00, ''lights off'') when the mice were in their most active period and had their highest body temperature.

Dexmedetomidine experiments

Prior to dexmedetomidine injection, animals with implanted temperature loggers were fitted with Neurologger 2A devices, and one hour of both baseline EEG/EMG data and core body temperature were recorded as reference. Dexmedetomidine (Tocris Bioscience) was dissolved in saline to make a final concentration of 50 μg per kg and delivered *i.p.* at ZT19 (12:00, ''lights-off''). Animals were placed back to their home cage immediately after injection for a further five-hour recording and the EEG/EMG data and core body temperature were simultaneously recorded. A six-hour baseline recording from the same mouse of its natural sleep-wake cycle and core body temperature between ZT18 to ZT24 (11:00-17:00, ''lights off'') were used for parallel comparison with the dexmedetomidine injection experiments.

Immunohistochemistry

Mice were fixed by transcardial perfusion with 4% paraformaldehyde (Thermo scientific) in PBS, pH 7.4 after deep anesthesia by pentobarbital (100 mg/kg body weight; *i.p.*). Brains were removed and preserved in 30% sucrose in PBS. 35 μm-thick coronal sections were sliced using a Leica VT1000S vibratome. Free-floating sections were washed three times in PBS each for 5 minutes, permeabilized in 0.4% Triton X-100 in PBS for 30 minutes, blocked by incubation with 5% normal goat serum (NGS) (Vector) plus 0.2% Triton X-100 in PBS for 1 hour (all performed in room temperature) and then incubated with antisera for GFP (rabbit, 1:1000, Life Technology, #A6455), mCherry (mouse, 1:1000, Clontech, #632543), or parvalbumin (rabbit, 1:1000, Abcam, #ab11427). Primary antisera were diluted in PBS with 2% NGS overnight at 4°C. The following day, primary antisera incubated sections were washed three times in PBS each for 10 minutes and subsequently incubated for 2 hours at room temperature in PBS with 2% NGS plus a dilution of an Alexa Fluor 488 goat anti-rabbit IgG (H+L) (1:1000, Molecular Probes, #A11034) or Alexa Fluor 594 goat anti-mouse IgG (H+L) (1:1000, Molecular Probes, #A11005). Sections were washed 4 times in PBS each for 10 minutes at room temperature and subsequently mounted on glass slides in Vectashield with mounting medium which contains DAPI (H-1200, Vector Laboratories).

QUANTIFICATION AND STATISTICAL ANALYSIS

Origin v8.6 and Prism6 were used for statistical analyses. Data collection and processing were randomized or performed in a counterbalanced way. The sample sizes and statistical test for each experiment are stated in the figure legends. Data are represented as the mean ± SEM. For the behavioral tests, two-tailed t tests, or one or two-way ANOVA were performed accordingly. p values are shown when they are less than 0.05 (*$p < 0.05$, **$p < 0.01$, ***$p < 0.001$, ****$p < 0.0001$). When multiple comparisons were made, the Holm-Bonferroni post hoc test was applied. Mice were excluded from the analysis if the histology did not confirm significant AAV transgene expression in the LPO area of the hypothalamus, or if the transgene expression had spread beyond the target region. Investigator were not blind to treatments.

DATA AND CODE AVAILABILITY

This study did not generate any datasets or code.

Current Biology, Volume *29*

Supplemental Information

Galanin Neurons Unite Sleep Homeostasis and α2-Adrenergic Sedation

Ying Ma, Giulia Miracca, Xiao Yu, Edward C. Harding, Andawei Miao, Raquel Yustos, Alexei L. Vyssotski, Nicholas P. Franks, and William Wisden

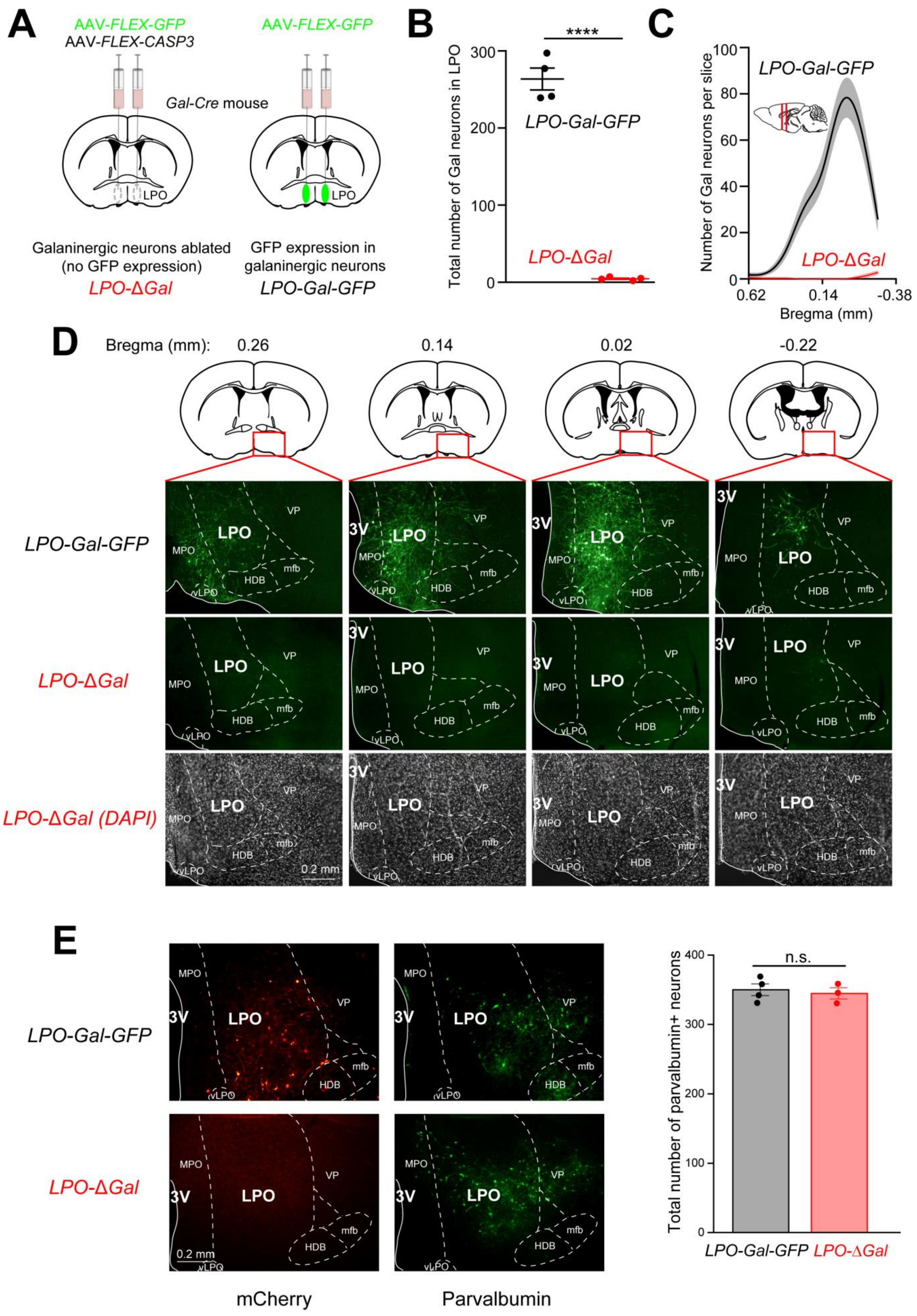
A
AAV-FLEX-GFP
AAV-FLEX-CASP3
AAV-FLEX-GFP
Gal-Cre mouse
LPO
LPO
Galaninergic neurons ablated
(no GFP expression)
LPO-ΔGal
GFP expression in
galaninergic neurons
LPO-Gal-GFP
B
****
Total number of Gal neurons in LPO
300
200
100
0
LPO-Gal-GFP
LPO-ΔGal
C
Number of Gal neurons per slice
100
80
60
40
20
0
LPO-Gal-GFP
LPO-ΔGal
0.62
0.14
-0.38
Bregma (mm)
D
Bregma (mm):
0.26
0.14
0.02
-0.22
LPO-Gal-GFP
LPO-ΔGal
LPO-ΔGal (DAPI)
LPO
MPO
VP
HDB
mfb
vLPO
3V
0.2 mm
E
LPO-Gal-GFP
LPO-ΔGal
mCherry
Parvalbumin
n.s.
Total number of parvalbumin+ neurons
400
300
200
100
0
LPO-Gal-GFP
LPO-ΔGal

Figure S1. Ablation of galanin neurons in the LPO of the hypothalamus. Related to Figure 1. (A) Galanin neurons in the LPO of *Gal-Cre* mice were ablated by bilaterally injecting AAV-*FLEX-CASP3*, together with AAV-*FLEX-GFP* to give *LPO-ΔGal* mice. Control animals (*LPO-Gal-GFP* mice) were injected with AAV-*FLEX-GFP* alone. (B) Most (~98%) galanin neurons in the LPO were ablated (****P<0.0001, paired two-tailed *t*-test). (C) Rostral-caudal distribution of galanin neurons before and after ablation. (D) Example images of galanin neurons in the LPO determined by GFP expression (top row) and after ablation (middle row). The bottom row shows the corresponding DAPI stains from the above immune-stained sections and alignment with the mouse brain atlas (The Allen Brain Atlas, http://www.brain-map.org). (E) Both *LPO-ΔGal* and *LPO-Gal-GFP* sections were stained for parvalbumin expression. Example expression patterns of parvalbumin at Bregma 0.14 were similar in both groups, and further quantification across the whole LPO area demonstrated LPO^{Parv} neurons were not affected by ablation of LPO^{Gal} neurons (unpaired two-tailed *t*-test). For this particular experiment, AAV-*FLEX-mCherry* was injected to both mouse groups instead of AAV-*FLEX-GFP* (to avoid antibody species cross reactivity). For the data in B, C and E, brain sections containing the LPO area from 4 individual mice were selected in each group, *i.e.* 4 brain sections for each coordinate in one group. 3V, the third ventricle; HDB, magnocellular preoptic nucleus; LPO, lateral preoptic; mfb, medial forebrain bundle; MPO, medial preoptic nucleus; vLPO, ventrolateral preoptic nucleus; VP, ventral pallidum. All error bars represent the SEM.

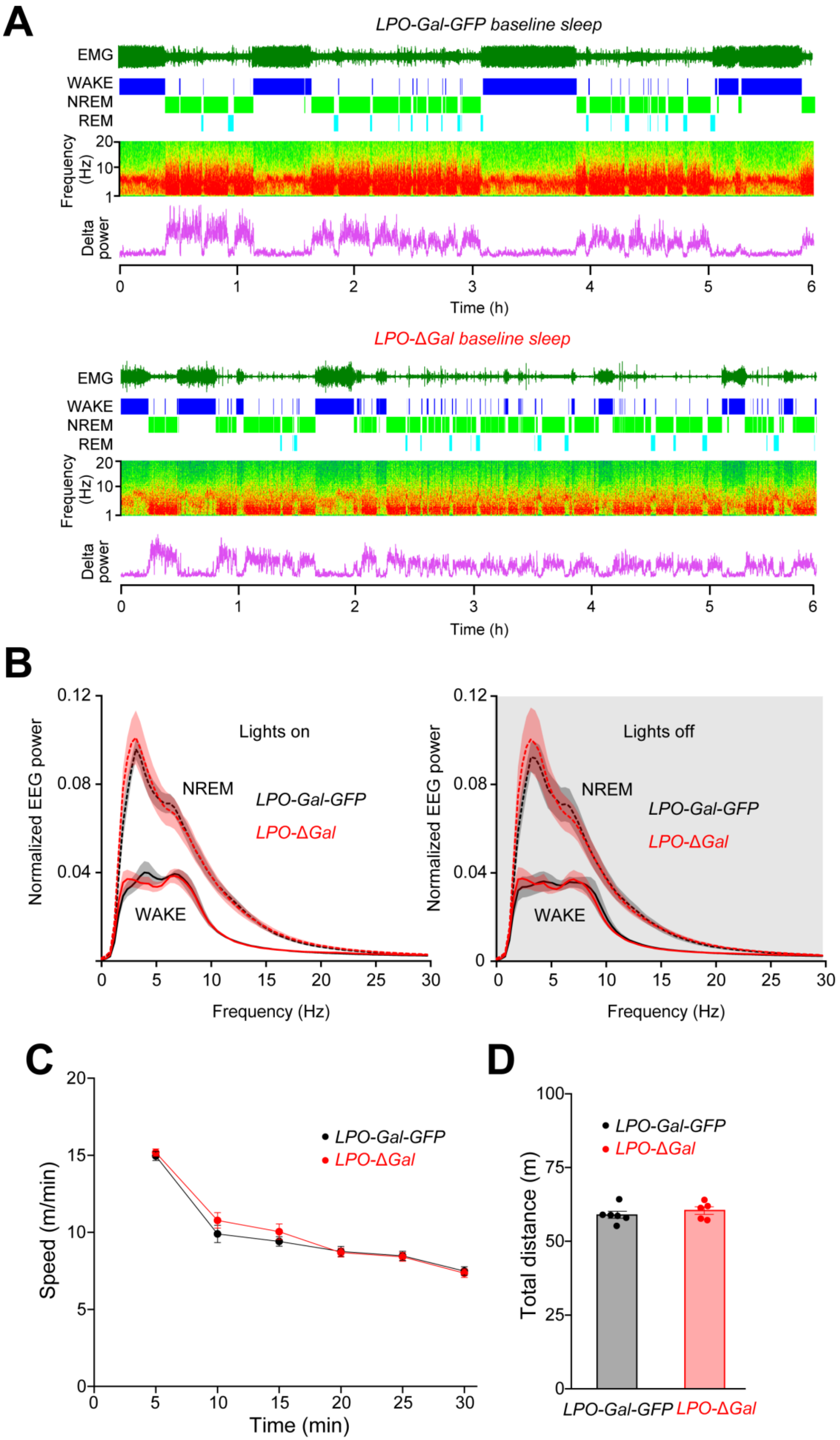

A
LPO-Gal-GFP baseline sleep
EMG
WAKE
NREM
REM
Frequency (Hz)
20
10
1
Delta power
0
1
2
3
4
5
6
Time (h)
LPO-ΔGal baseline sleep
EMG
WAKE
NREM
REM
Frequency (Hz)
Delta power
Time (h)
B
Normalized EEG power
0.12
0.08
0.04
Lights on
NREM
WAKE
LPO-Gal-GFP
LPO-ΔGal
Frequency (Hz)
0
5
10
15
20
25
30
Lights off
NREM
WAKE
LPO-Gal-GFP
LPO-ΔGal
Frequency (Hz)
C
Speed (m/min)
20
15
10
5
0
LPO-Gal-GFP
LPO-ΔGal
Time (min)
D
Total distance (m)
100
75
50
25
0
LPO-Gal-GFP
LPO-ΔGal

Figure S2. Sleep and locomotion in *LPO-Gal-GFP* mice and *LPO-∆Gal* mice. Related to Figure 1 and Figure 2. (A) Examples of EEG and EMG raw data and vigilance-state scoring for *LPO-Gal-GFP* mice and *LPO-∆Gal* mice (from ZT0 to ZT6). (B) EEG power normalized such that the area under the curve was unity during the waking state. There were no significant differences in the EEG power in either the WAKE state or NREM state between *LPO-Gal-GFP* mice and *LPO-∆Gal* mice (*LPO-Gal-GFP*, n=8; *LPO-∆Gal*, n=6). (C, D) The locomotion and speed of *LPO-Gal-GFP* (black; n=6) and *LPO-∆Gal* (red; n=5) mice were determined for 30 mins with an open field test in the “lights off” period (around ZT 18). No differences between the two groups were found (P>0.93, repeated measures one-way ANOVA with post hoc test for C; P>0.98, unpaired two-tailed t-test for D). All error bars represent the SEM.

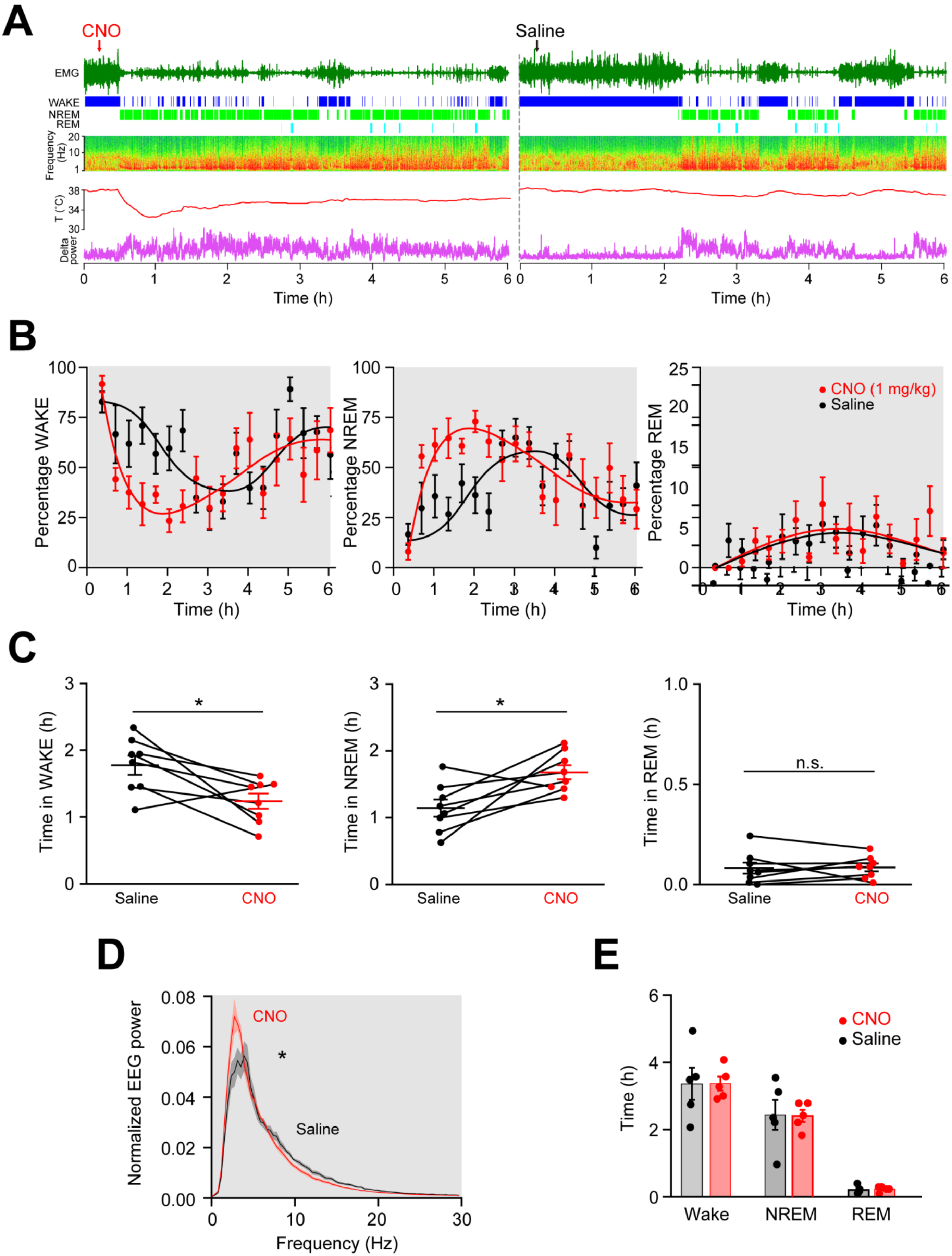

A
CNO
Saline
EMG
WAKE
NREM
REM
Frequency (Hz)
T (°C)
Delta power
Time (h)
B
Percentage WAKE
Percentage NREM
Percentage REM
CNO (1 mg/kg)
Saline
Time (h)
C
Time in WAKE (h)
Time in NREM (h)
Time in REM (h)
*
n.s.
Saline
CNO
D
Normalized EEG power
CNO
Saline
Frequency (Hz)
E
Time (h)
CNO
Saline
Wake
NREM
REM

Figure S3. Selective chemogenetic activation of LPO^{Gal} neurons by CNO increased NREM sleep. Related to Figure 1. (A) Examples of EEG and EMG raw data and vigilance-state scoring, core body temperature and delta power for *LPO-Gal-*hM_3D_q mice following CNO (1 mg/kg) (left) or saline (right) *i.p.* injection. (B) The percentage of WAKE reduced and the percentage of NREM increased following CNO injection compared with control saline injection. The percentage of time in REM did not change (n=8). (C) Total time in WAKE, NREM and REM over the three hours following injection. (*P<0.05, paired two-tailed t-test; n=8). (D) The delta power of the CNO-induced NREM-like sleep had a significantly higher power than that of NREM sleep after saline injection (at the same time of injection) (*P<0.05, paired two-tailed t-test; n=8). Traces over the first 3 hours post injection. (E) Control for off-target effects: CNO (1 mg/kg) injected *i.p.* into *Gal-Cre* mice (that had not been injected with AAV-*FLEX-*hM_3D_q) did not induce NREM sleep above normally occurring amounts over the next 6 hours (compared with *Gal-Cre* mice that had received saline injections). All error bars represent the SEM.

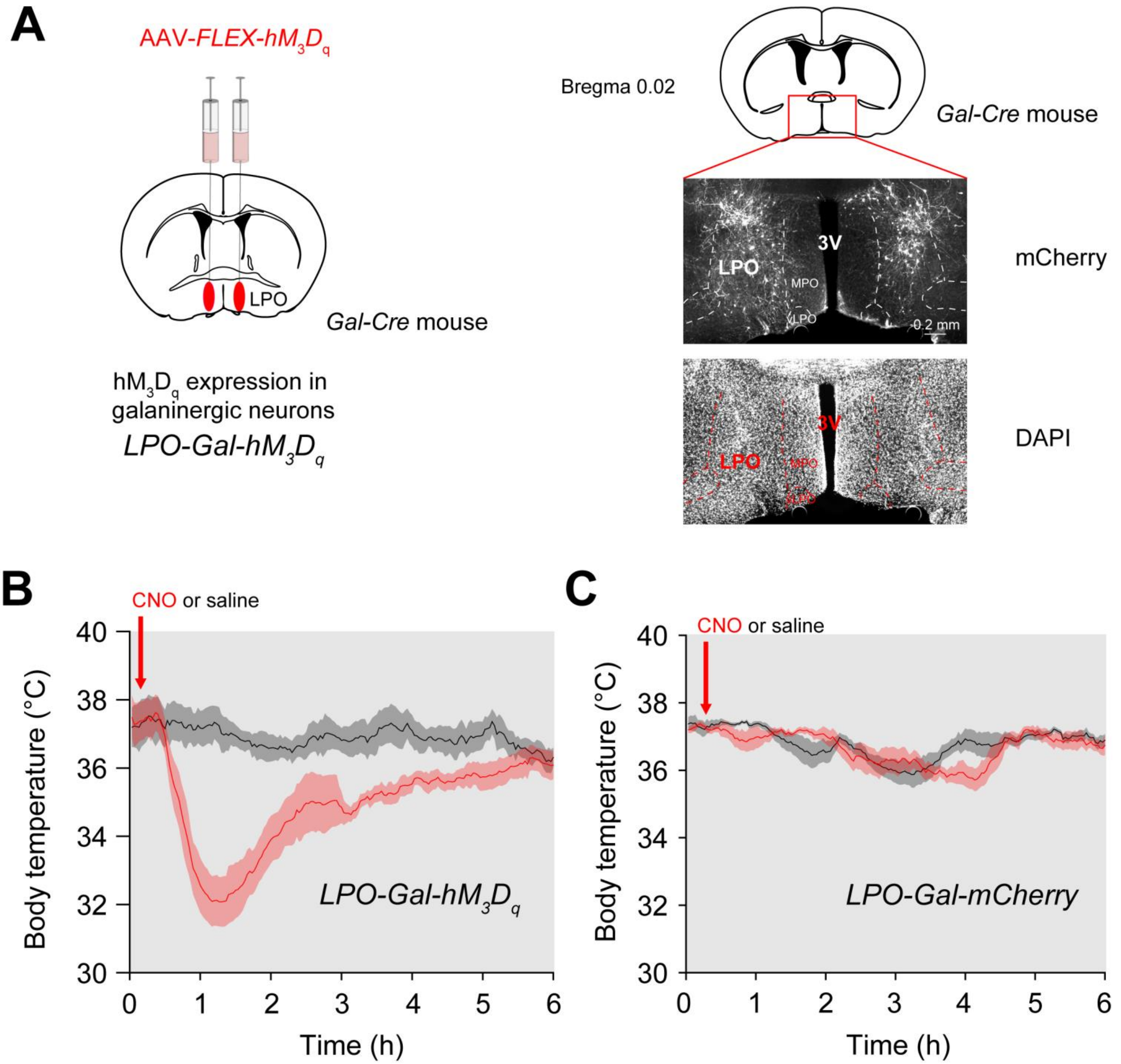

A
AAV-FLEX-hM3Dq
LPO
Gal-Cre mouse
hM3Dq expression in galaninergic neurons
LPO-Gal-hM3Dq
Bregma 0.02
Gal-Cre mouse
3V
LPO
MPO
VLPO
0.2 mm
mCherry
DAPI
B
CNO or saline
Body temperature (°C)
Time (h)
LPO-Gal-hM3Dq
C
CNO or saline
Body temperature (°C)
Time (h)
LPO-Gal-mCherry

Figure S4. Selective chemogenetic activation of LPO^{Gal} neurons induces hypothermia. Related to Figure 2. (A) Galanin neurons in the LPO of *Gal-Cre* mice were made selectively sensitive to CNO by bilaterally injecting AAV-*FLEX-hM$_3$D$_q$* to generate *LPO-Gal-hM$_3$D$_q$* mice. The hM$_3$D$_q$ receptor is fused to the mCherry protein, permitting visualization of receptor expression by immunohistochemistry with mCherry antibodies. The corresponding DAPI staining is shown below. Receptor expression was largely confined to the LPO area. 3V, the third ventricle; LPO, lateral preoptic nucleus; MPO, medial preoptic nucleus; vLPO, ventrolateral preoptic nucleus. (B) CNO (1 mg/kg) but not saline injection (*i.p.*) induced a strong acute hypothermia in *LPO-Gal-hM$_3$D$_q$* mice lasting several hours (*n*=5). (C) Control for off-target effects. CNO (1 mg/kg) injected (*i.p.*) into *Gal-Cre* mice (that had not been injected with AAV-*FLEX-hM$_3$D$_q$*) did not induce hypothermia compared with *Gal-Cre* mice that had received saline injections (*n*=5). All error bars represent the SEM.
